# Small-Molecule Inhibitor of Flaviviral NS3-NS5 Interaction with Broad-Spectrum Activity and Efficacy *In Vivo*

**DOI:** 10.1128/mbio.03097-22

**Published:** 2023-01-09

**Authors:** Marta Celegato, Mattia Sturlese, Vivian Vasconcelos Costa, Marta Trevisan, Angélica Samer Lallo Dias, Ingredy Beatriz Souza Passos, Celso Martins Queiroz-Junior, Lorenzo Messa, Annagiulia Favaro, Stefano Moro, Mauro Martins Teixeira, Arianna Loregian, Beatrice Mercorelli

**Affiliations:** a Department of Molecular Medicine, University of Padua, Padua, Italy; b Molecular Modeling Section (MMS), Department of Pharmaceutical and Pharmacological Sciences, University of Padua, Padua, Italy; c Departamento de Morfologia, ICB, Universidade Federal de Minas Gerais, Belo Horizonte, Brazil; d Departamento de Bioquímica e Imunologia, ICB, Universidade Federal de Minas Gerais, Belo Horizonte, Brazil; La Jolla Institute for Allergy and Immunology; Washington University School of Medicine

**Keywords:** flavivirus, NS3-NS5 interaction, dissociative inhibitors, antiviral drugs, dengue virus, West-Nile virus, Zika virus, antiviral agents

## Abstract

Every year, dengue virus (DENV) causes one hundred million infections worldwide that can result in dengue disease and severe dengue. Two other mosquito-borne flaviviruses, i.e., Zika virus (ZIKV) and West Nile virus (WNV), are responsible of prolonged outbreaks and are associated with severe neurological diseases, congenital defects, and eventually death. These three viruses, despite their importance for global public health, still lack specific drug treatments. Here, we describe the structure-guided discovery of small molecules with pan-flavivirus antiviral potential by a virtual screening of ~1 million structures targeting the NS3-NS5 interaction surface of different flaviviruses. Two molecules inhibited the interaction between DENV NS3 and NS5 *in vitro* and the replication of all DENV serotypes as well as ZIKV and WNV and exhibited low propensity to select resistant viruses. Remarkably, one molecule demonstrated efficacy in a mouse model of dengue by reducing peak viremia, viral load in target organs, and associated tissue pathology. This study provides the proof of concept that targeting the flaviviral NS3-NS5 interaction is an effective therapeutic strategy able to reduce virus replication *in vivo* and discloses new chemical scaffolds that could be further developed, thus providing a significant milestone in the development of much awaited broad-spectrum antiflaviviral drugs.

## INTRODUCTION

Dengue virus (DENV) (the most prevalent arbovirus), Zika virus (ZIKV), and West Nile virus (WNV) are RNA viruses belonging to the *Flaviviridae* family and are transmitted to humans by mosquitoes. Each of the four different DENV serotypes (DENV-1 to DENV-4) can cause dengue, a systemic disease characterized by different clinical manifestations (1–3). Almost 400 million DENV infections occur annually, of which 500,000 cases/year develop severe dengue that can be fatal (1–3). More than 3 billion people (>40% of the world human population) live in at-risk geographical areas, but the risk may be even higher (4). ZIKV and WNV are endemic in tropical and subtropical areas but are responsible for outbreaks in other regions, such as in the United States and Europe (1, 5). ZIKV infection is mostly asymptomatic and self-limiting, but in the case of fetal infection, it can cause ZIKV congenital syndrome, and neurological complications have also been reported in adults, such as Guillain-Barré syndrome (6–8). WNV infects both domestic animals and humans, causing different diseases ranging from a mild febrile state to severe neurological damage leading to encephalitis and death (9). In the near future, the WHO and governments will have to deal with the pandemic potential of existing and emerging flaviviruses in countries where this problem has not yet been addressed (5). To date, vector control strategies have failed, no specific approved drug for dengue or other mosquito-borne flaviviruses is available, and fully protective and safe vaccines are still lacking (3, 10). Thus, the development of effective therapeutic strategies is a paramount priority for global health management. The availability of drugs with pan-flavivirus antiviral activity could be crucial in the management of associated diseases and be of particular benefit in the case of coinfections with more than one flavivirus, epidemic outbreaks, and (particularly for DENV) reinfections (11).

Traditionally, drug discovery campaigns for flaviviruses have been focused on direct targeting of the two sole viral enzymes, i.e., the viral RNA-dependent RNA polymerase (RdRP) NS5 or protease NS2B/NS3 (12–14); however, candidate drugs that act by these mechanisms have not yet reached the clinic and, experimentally, were reported to be associated with toxicity and emergence of resistance (15, 16). Thus, other approaches have been explored, such as targeting either other viral proteins (17, 18) or the host (19–21). Blocking protein-protein interactions (PPI) essential for virus replication has proven to be a successful alternative antiviral strategy (22–24). In the case of flaviviruses, the interaction between the NS3 helicase (NS3-hel) domain and NS5-RdRp domain is essential for replication, and mutations that block their association have a detrimental effect on virus replication (25–27); thus, it represents an attractive target for a dissociative therapeutic strategy.

Taking advantage of the crystal structures of NS5 of different flaviviruses (i.e., DENV, ZIKV, and WNV), we generated a computational model to simulate the molecular dynamics of NS3-NS5 interaction of different flaviviruses and performed a virtual screening of ~1 million small-molecule structures in order to identify potential NS3-NS5 interaction inhibitors with pan-dengue and broad-spectrum antiflavivirus activity. The structure-guided approach led to the discovery of two structurally different small molecules able to block NS3-NS5 interaction *in vitro* and the replication of different flaviviruses in infected cells, and characterized by a low propensity to select resistant viruses. Moreover, one of these molecules proved, for the first time, efficacy *in vivo* in a mouse model of dengue infection.

## RESULTS

### Virtual screening for small molecules targeting flaviviral NS3-NS5 interface.

**FIG 1:**
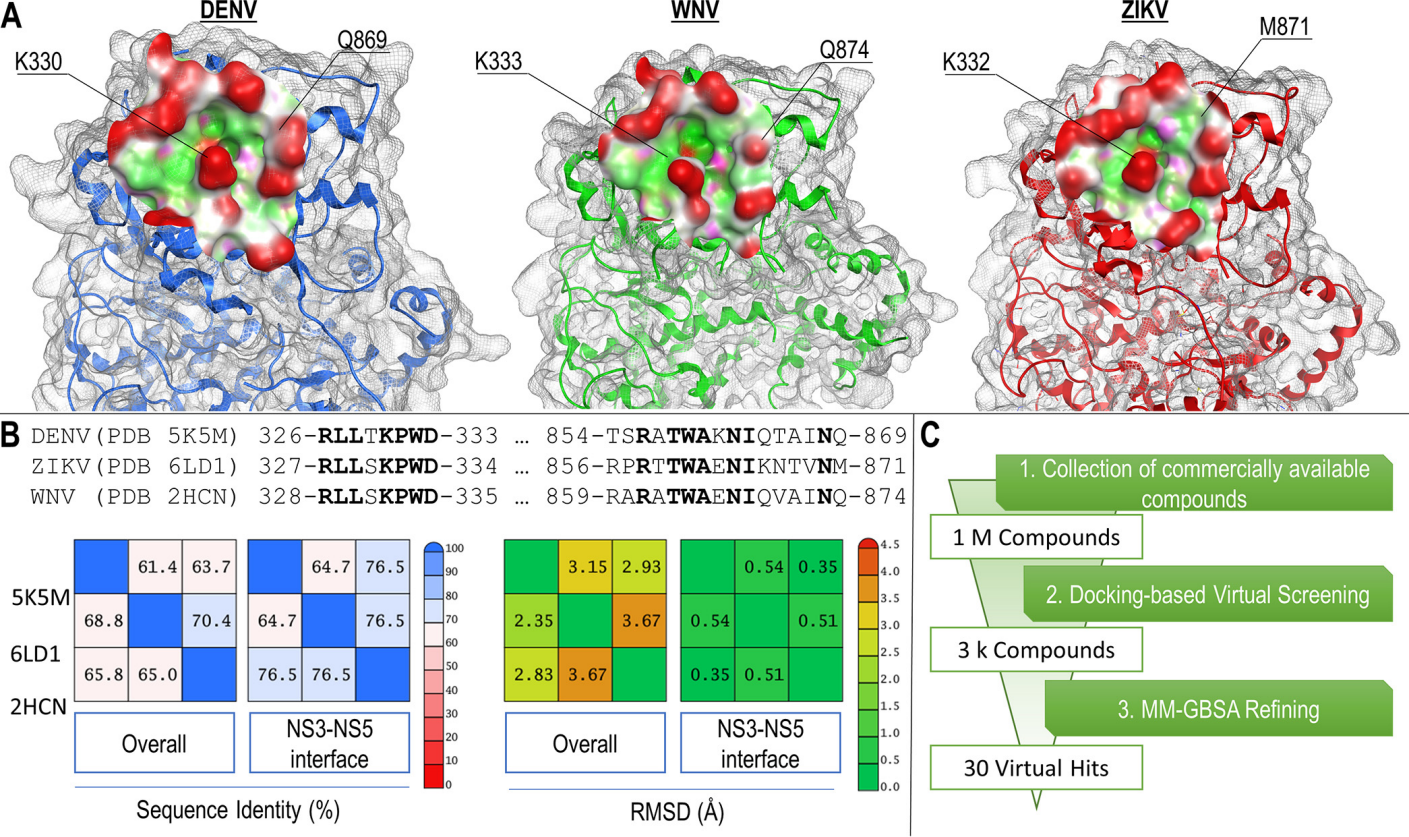
Structure-guided virtual screening of inhibitors of NS3-NS5 interaction. (A) Analysis of NS3-NS5 interaction surface on NS5 of DENV, ZIKV, and WNV (PDB accession numbers are in panel B). The residues of NS5 involved in the interaction with NS3 are indicated. (B) Analysis of the similarity between flavivirus NS5 cavity B in terms of residue conservation (% of sequence identity) and the average distance between the atoms (RMSD, expressed in angstrom, Å) for both NS5 and the NS3-NS5 interface. Conserved residues are highlighted in bold. (C) Schematic workflow of the experimental approach followed for the virtual screening. (MM-GBSA, molecular mechanics energies combined with generalized Born and surface area continuum solvation, i.e., the method used to estimate the free energy of the binding of small molecules to NS5).

With the aim of identifying small molecules able to bind at the flaviviral NS3-NS5 interface, we focused on the pocket surrounding a residue of NS5 crucial for the interaction with NS3, i.e., K330 in DENV-2 (Fig. 1A) (25). The selected pocket showed good conservation in all DENV serotypes, ZIKV, and WNV, with high sequence identity (61 to 70%) as well as remarkable structural similarity with a root mean square deviation (RMSD) in the range of 0.35 to 0.54 Å for the backbone of the pocket residues (Fig. 1B). Moreover, the shape of the cavity around the conserved lysine residue (K330 in DENV, K332 in ZIKV, K333 in WNV) was well conserved. Given these observations, we decided to use DENV NS5 structure for the virtual screening (VS) (Fig. 1C). First, we generated a library containing ~1 million compounds from commercially available collections of chemical entities, which was then subjected to a docking-based VS using a plants docking engine (28). Ten million ligand conformations (i.e., 10 for each compound) resulted from the VS and were then filtered to remove those with unfavorable steric clashes or high (adverse) electrostatic potential energy. Then, the 3,000 best-scoring compounds were promoted to a further ranking procedure leading to 30 virtual hits (see Fig. S1A in the supplemental material). All of the selected hits were then docked on NS5 of WNV and ZIKV. All of the virtual hits showed the ability to assume a U shape around the central conserved lysine and, hence, to possibly act as inhibitors of NS3-NS5 interactions of all three flaviviruses. The poses of the 30 hit compounds are described in Movie S1 in the supplemental material.

10.1128/mbio.03097-22.1 FIG S1 Chemical structures of the compounds considered in the study. (A) Chemical structures of the 30 hit compounds identified by virtual screening. (B) Chemical structures of the analogs of hit compound C-9. Download FIG S1, DOCX file, 0.4 MB. Copyright © 2023 Celegato et al. 2023 Celegato et al. https://creativecommons.org/licenses/by/4.0/ This content is distributed under the terms of the Creative Commons Attribution 4.0 International license.

10.1128/mbio.03097-22.9 MOVIE S1 Poses of the 30 hit compounds identified by VS. The binding mode of the best pose selected for each of the 30 hits identified by virtual screening on DENV-2 NS5 are reported. The molecular surface colored according to its electrostatics nature (red to blue palette indicate positive to negative charge). Most relevant protein residues are reported in sticks while the ligands are in ball and stick representation. Download Movie S1, AVI file, 0.8 MB. Copyright © 2023 Celegato et al. 2023 Celegato et al. https://creativecommons.org/licenses/by/4.0/ This content is distributed under the terms of the Creative Commons Attribution 4.0 International license.

### Biological profile of hit compounds against DENV.

**FIG 2:**
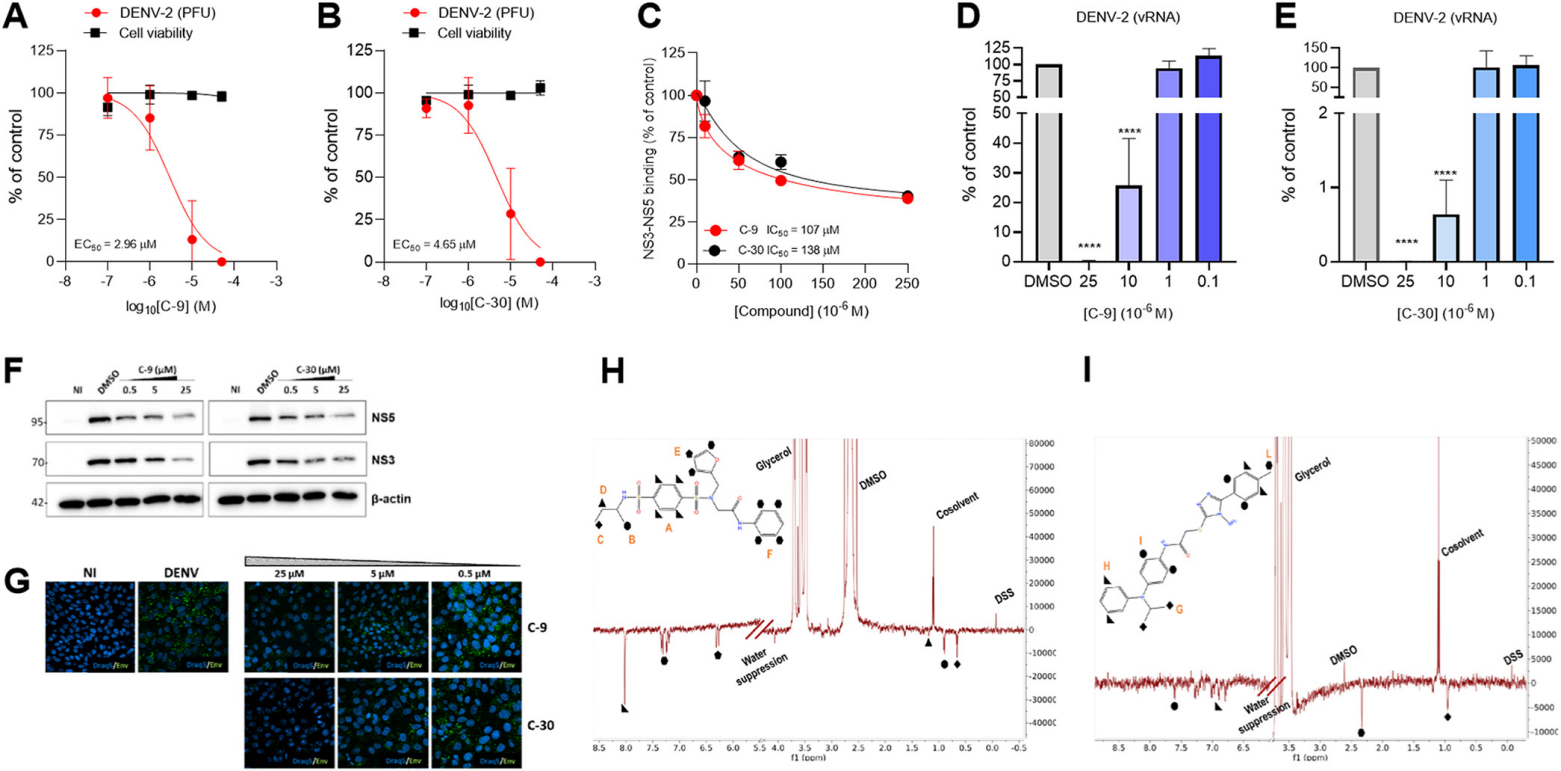
Antiviral profile of hit compounds C-9 and C-30. (A and B) Concentration-dependent inhibition of DENV-2 replication in infected Vero cells and effects on cell viability in uninfected cells at 72 h upon treatment with C-9 and C-30, respectively. (C) Concentration-dependent inhibition of NS3-NS5 binding *in vitro*. (D and E) Concentration-dependent inhibition of DENV-2 RNA synthesis by C-9 and C-30 as determined by RT-qPCR in infected Vero cells at 72 h p.i. Graphs represent the mean ± standard deviation (SD) of *n* = 3 independent experiments in duplicate. Data were analyzed by a one-way analysis of variance (ANOVA) followed by Dunnett’s multiple comparison test. ****, *P* < 0.0001 compared to control (infected, DMSO-treated sample). (F) Inhibition of NS3 and NS5 expression. Vero cells were infected with DENV-2 and treated with different concentrations of hit compounds C-9 and C-30 or 0.1% DMSO as a control. Whole cell lysates obtained from cells collected at 56 h p.i. were then analyzed by Western blotting with antibodies recognizing DENV-2 NS3 and NS5. β-actin was used as a loading control. Molecular masses in kDa are indicated on the left. Images of a representative experiment are shown. (G) Analysis of Env protein expression during DENV-2 infection. Viral Env protein expression was detected at 56 h p.i by immunofluorescence analysis in uninfected Vero cells (NI) and in Vero cells infected with DENV-2 (DENV) and treated with either 0.1% DMSO or different concentrations of C-9 and C-30 hit compounds. DRAQ5 was used to stain the cell nuclei. Images of a representative experiment are shown. (H and I) Interaction of hit compounds with DENV NS5 RdRP. WaterLOGSY spectra of 50 μM C-9 and C-30, respectively, in the presence of NS5 (0.5 μM). The assignment of the signals is reported on the 2D structure of the molecules.

We first investigated the effects of the 30 hit compounds against DENV. Among these, C-9 and C-30 were the most promising, since they inhibited DENV-2 replication in a concentration-dependent manner without showing cytotoxicity and having a very favorable Selectivity Index (SI) (Fig. 2A and B; see Fig. S2 and Table S1 in the supplemental material). The compounds could be tested only up to 250 μM, since they tend to form precipitates at higher concentrations. In parallel, to investigate whether the 30 small molecules selected by VS could actually inhibit the binding between DENV-2 NS3 and NS5 *in vitro*, we developed an enzyme-linked immunosorbent assay (ELISA)-based assay to measure the interaction of NS3-Hel with NS5-RdRP (see Fig. S3A to C in the supplemental material). We performed concentration-response analyses of the inhibition of the NS3-NS5 interaction for the 30 hit compounds by ELISA, and 14 compounds caused a concentration-dependent reduction in absorbance (see Fig. S3D and Table S1). Only C-9 and C-30 were able to inhibit both the replication of DENV-2 in infected cells without affecting cell viability and the NS3-NS5 interaction (Fig. 2A to C; see also Table S1). The inhibition of the NS3-NS5 interaction was specific because the addition of an unrelated dissociative small molecule able to inhibit the interaction between influenza virus PA and PB1 RNA polymerase subunits (23) did not affect the NS3-NS5 interaction (Fig. S3C), and both compounds did not interfere with the unrelated PA-PB1 interaction (Fig. S3E); thus, these compounds were selected for follow-up studies.

10.1128/mbio.03097-22.2 FIG S2 Concentration-dependent inhibition of DENV-2 NGC replication by selected hit compounds. Plaque reduction assays were performed in Vero cells infected with DENV-2 NGC strain and treated with different doses (from 0.1 to 50 μM) of the indicated test compounds. Graph represents mean ± SD of *n* ≥ 3 independent experiments in duplicate. Reported are concentration-response curves for 9 out of 30 hit compounds. Download FIG S2, DOCX file, 0.1 MB. Copyright © 2023 Celegato et al. 2023 Celegato et al. https://creativecommons.org/licenses/by/4.0/ This content is distributed under the terms of the Creative Commons Attribution 4.0 International license.

10.1128/mbio.03097-22.3 FIG S3 DENV-2 ELISA-based NS3-NS5 interaction assay. (A) Concentration-dependent increase in absorbance when increasing amounts of 6His-NS5(279-900), but not of unrelated 6His-IE2(290-579), were added to wells coated with 300 ng of 6His-NS3(177-618). (B) The addition to the ELISA NS3-NS5 mixture of increasing amounts of free 6His-NS3(177-618) resulted in a concentration-dependent reduction in absorbance due to competitive binding to unbound NS5. (C) Addition to the ELISA NS3-NS5 mixture of increasing amounts of Flu-1, a previously described dissociative inhibitor of influenza virus PA-PB1 interaction, did not affect NS3-NS5 binding. Data shown represent the mean ± SD of *n* = 3 independent experiments. (D) Concentration-dependent inhibition of NS3-NS5 interaction in ELISA-based assays *in vitro*. Increasing concentrations of the indicated compounds were added together with 200 ng of 6His-NS5(279-900) to wells coated with 300 ng of 6His-NS3(177-618). Binding of 6His-NS5(279-900) was quantified as described in Fig. S3. Data shown represent the mean ± SD of *n* = 3 independent experiments. Reported are dose-response curves for 14 out of 30 hit compounds. (E) Absence of inhibitory effects of C-9 and C-30 on influenza virus RNA polymerase subunits PA-PB1 interaction *in vitro*. Download FIG S3, DOCX file, 0.1 MB. Copyright © 2023 Celegato et al. 2023 Celegato et al. https://creativecommons.org/licenses/by/4.0/ This content is distributed under the terms of the Creative Commons Attribution 4.0 International license.

10.1128/mbio.03097-22.5 TABLE S1 Antiviral activity and cytotoxicity of hit compounds against DENV-2. Download Table S1, DOCX file, 0.01 MB. Copyright © 2023 Celegato et al. 2023 Celegato et al. https://creativecommons.org/licenses/by/4.0/ This content is distributed under the terms of the Creative Commons Attribution 4.0 International license.

To further characterize the antiviral profile of C-9 and C-30, we assessed their effects on viral RNA synthesis and on viral protein expression during DENV-2 infection. As reported in Fig. 2D to G, a significant reduction of viral RNA synthesis and of essential NS3, NS5, and Env proteins was observed when infected cells were treated with different concentrations of both compounds. Altogether, these results indicated that hit compounds C-9 and C-30 are able to reduce viral RNA synthesis in infected cells and inhibit the expression of different essential viral proteins in the context of DENV-2 infection.

### Biophysical characterization of the interaction of hit compounds to NS5 RdRP by NMR.

To further demonstrate that the hit compounds directly bind NS5 protein, we acquired a WaterLOGSY nuclear magnetic resonance (NMR) experiment, a sensitive and widely used ligand-observed experiment for detection of the interaction between a ligand and a protein. This experiment clearly proved that both C-9 and C-30 bind to NS5 RdRP protein. In fact, as shown in Fig. 2H and I, the signals of the ligands assume the same negative sign as the protein in the presence of NS5, indicating that the ligands acquired the protein magnetization as intermolecular nuclear Overhauser effect (NOE) because of the formation of the protein-ligand complex. Differently, the signals arising from the glycerol, dimethyl sulfoxide (DMSO), cosolvent, and the internal standard dimethyl sulfoxide (DSS) present the opposite (positive) phase, thus indicating that they do not interact with NS5. Moreover, the negative intensity of the signals of the aromatic protons of the 1,4-benzenedisulfonamide (group A) of C-9 (Fig. 2H) are stronger than that of the signals of the other moieties, suggesting that this portion of the molecule is more in contact with the protein and less exposed to the solvent. These experimental findings nicely agree with the predicted binding pose originating from the computational studies. The signals of C-30 hit compound show similar negative intensities (Fig. 2I), suggesting that the ligand is more exposed to the solvent or that it could undergo larger fluctuation in the bound state.

### Pan-serotype anti-DENV activity of hit compounds C-9 and C-30.

**FIG 3:**
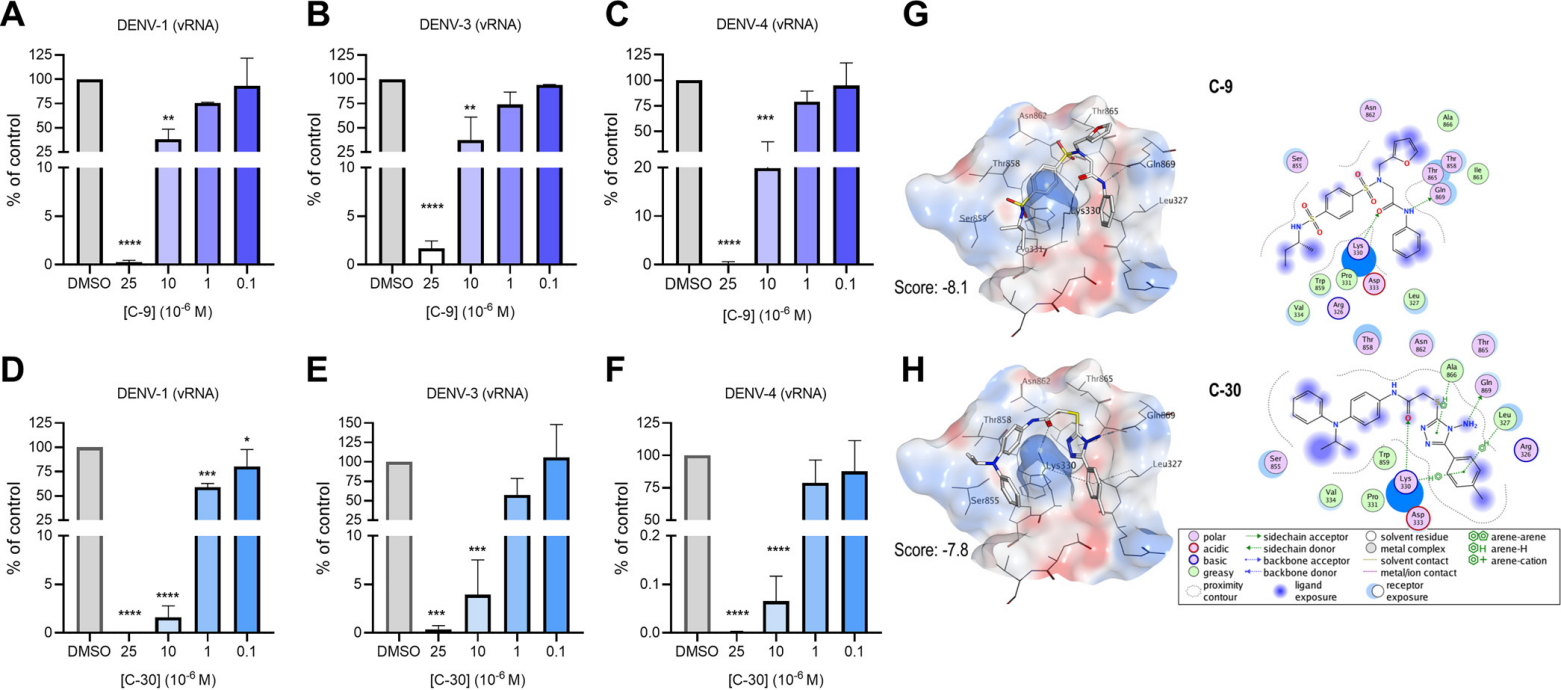
Pan-serotype anti-DENV profile of hit compounds C-9 and C-30. (A to C) Concentration-dependent inhibition of DENV-1 (TH-Sman), DENV-3 (H87), and DENV-4 (H-241) viral RNA synthesis in Vero cells treated with different concentrations of C-9 as determined by RT-qPCR at 72 h p.i. (D to F) Concentration-dependent inhibition of DENV-1, DENV-3, and DENV-4 viral RNA synthesis in Vero cells treated with different concentrations of C-30 as determined by RT-qPCR at 72 h p.i. Graphs represent the mean ± SD of *n* = 3 independent experiments in duplicate. Data were analyzed by a one-way ANOVA followed by Dunnett’s multiple comparison test. ****, *P* < 0.0001; ***, *P* < 0.001; **, *P* < 0.005 compared to control (infected, DMSO-treated sample). (G) Binding mode of C-9 with DENV-2 NS5. (Left) Best pose for C-9 in DENV-2 NS5. Selected residues are reported in sticks mode. (Right) Two-dimensional (2D) ligand-interaction diagram of C-9 with NS5 cavity reporting the most relevant residues for binding. (H) Binding mode of C-30 with DENV-2 NS5. (Left) Best pose for C-30 in DENV-2 NS5. Selected residues are reported in sticks mode. (Right) 2D ligand-interaction diagram of C-30 with NS5 cavity reporting the most relevant residues or binding. In panels G and H, the final score (MM-GB/VI) after the flexible refinement is reported for each pose.

Dengue disease can be caused by four different DENV serotypes (DENV-1 to DENV-4) that differ in epidemiology, endemic potential, and association with severe dengue (1). Thus, we assessed the pan-serotype antiviral activity of hit compounds C-9 and C-30 by testing them against different DENV serotypes. As reported in Fig. 3A to F, both C-9 and C-30 inhibited the replication of all DENV serotypes in a concentration-dependent manner, with effective concentrations at half-maximal response (EC_50_) comparable among the different serotypes (Table 1), thus showing pan-serotype anti-DENV activity.

**TABLE 1:** Antiviral activity of C-9 and C-30 against different flaviviruses

Flavivirus (strain)	Antiviral activity, EC_50_^*a*^ (μM) (95% CI)^*b*^	
	C-9	C-30
DENV-1 (TH-Sman)	3.60 (0.57**–**11.9)	1.32 (0.78**–**2.17)
DENV-2 (New Guinea C)	2.44 (1.39**–**4.28)	3.66 (1.78**–**7.41)
DENV-2 (TH-36)	1.67 (0.84**–**3.04)	1.24 (0.60**–**2.42)
DENV-3 (H-87)	5.74 (1.62**–**17.62)	1.64 (0.55**–**4.66)
DENV-4 (H-241)	3.22 (1.41**–**6.90)	2.30 (1.12**–**4.54)
ZIKV (PRVABC59)	2.10 (1.27**–**3.49)	1.59 (1.05**–**2.38)
ZIKV (clinical isolate)	2.10 (1.04**–**4.12)	3.49 (1.47**–**8.25)
WNV (NY99)	1.66 (0.88**–**2.87)	1.97 (1.28**–**2.96)

a Effective concentration at half-maximal response, the compound concentration that inhibits 50% of viral RNA synthesis at 72 h p.i., as determined by RT-qPCR in supernatant of infected Vero cells against all viruses except for DENV-2 TH-36 strain, ZIKV, and WNV for which plaque reduction assays in infected Vero cells were performed. Reported values derived from *n *≥ 3 independent experiments in duplicate.

b CI, Confidence interval (95% profile likelihood, determined with GraphPad Prism 8.0 software).

To support these results at a molecular level, we evaluated the conservation of the residues predicted to be involved in the interactions of both compounds in the cavity B of NS5. Interestingly, these are the same residues conserved across all DENV serotypes and responsible for the interaction with NS3 (16) (Fig. 3G and H). In detail, both C-9 and C-30 are accommodated in the region of NS5 where NS3-interacting residues are located (i.e., cavity B residues L327, K330, W859, A866, and G869). C-9 and C-30 share an amidic moiety oriented toward K330 that plays a crucial role by establishing a hydrogen bond with this residue and guaranteeing, at the same time, an adequate size to be hosted in such a restricted cavity. The same amide group in C-9 is able to act as a hydrogen bond donor for Q869, while this interaction in C-30 is absent, since the amide is differently oriented. However, in this interaction, the hydrogen bond is restored by the amino-triazole ring. The different orientation of the amide in C-30 facilitates the electrostatic interaction of this group with N862. In C-9, the interaction of these residues is maintained thanks to the tertiary sulfonamide. Both compounds share an interaction pattern by hydrophobic contacts with V324, L327, P331 (Fig. 3G and H). Since all of the predicted interactions of NS5 with the two compounds involve amino acids conserved among all DENV serotypes, this most likely results in the pan-dengue antiviral activity that we observed for both hit compounds. To confirm these docking predictions, we attempted to obtain a cocrystal structure of C-9 in complex with NS5 of DENV-2, but unfortunately, it failed. We reasoned that the binding of C-9 to NS5, although sufficient to block NS3-NS5 interaction *in vitro* and in infected cells, might be transient, thus precluding cocrystallization.

### C-9 and C-30 show low propensity to select viruses with altered susceptibility.

To validate NS3-NS5 interaction as a target of C-9 and C-30, as well as to evaluate the barrier to drug resistance, we attempted to raise resistant viral mutants by following an approach similar to others previously described (17, 29). Briefly, DENV-2 NGC in quadruplicate was serially passaged in the presence of increasing concentrations of C-9 and C-30 for a total of 10 passages (P10) (see Table S2A in the supplemental material). To exclude possible mutations unrelated to the hit compounds’ mechanism of action, we also serially passaged DENV-2 in the presence of DMSO in parallel. As reported in Fig. S4 in the supplemental material, after 10 passages, we did not observe altered sensitivity of the viruses to both hit compounds compared to the DMSO-passaged DENV-2 virus. Thus, both C-9 and C-30 demonstrated a high barrier to drug-induced resistance.

10.1128/mbio.03097-22.4 FIG S4 Concentration-dependent inhibition of DENV-2 clones passaged in the presence of C-9 and C-30. Plaque reduction assays were performed in Vero cells infected with either DENV-2 NGC P10 strain passaged in DMSO or different DENV-2 P10 viruses (clones A to D) extensively passaged in the presence of increasing concentrations of hit compounds C-9 (A) and C-30 (B) (please refer also to Table S2A in the supplemental material). Vero cells were infected with the different viruses and then treated with increasing doses (from 0.1 to 25 μM) of the indicated test compounds. Graphs represent the mean of *n* = 2 independent experiments in duplicate. Download FIG S4, DOCX file, 0.1 MB. Copyright © 2023 Celegato et al. 2023 Celegato et al. https://creativecommons.org/licenses/by/4.0/ This content is distributed under the terms of the Creative Commons Attribution 4.0 International license.

10.1128/mbio.03097-22.6 TABLE S2 Additional supplementary information about the selection of resistant viruses, oligonucleotides, and antibodies. (A) Scheme of the DENV-2 resistant strains isolation procedure. (B) Oligonucleotides used in this study. (C) Antibodies used in this study. Download Table S2, DOCX file, 0.01 MB. Copyright © 2023 Celegato et al. 2023 Celegato et al. https://creativecommons.org/licenses/by/4.0/ This content is distributed under the terms of the Creative Commons Attribution 4.0 International license.

### Antiviral activity against other mosquito-borne flaviviruses.

**FIG 4:**
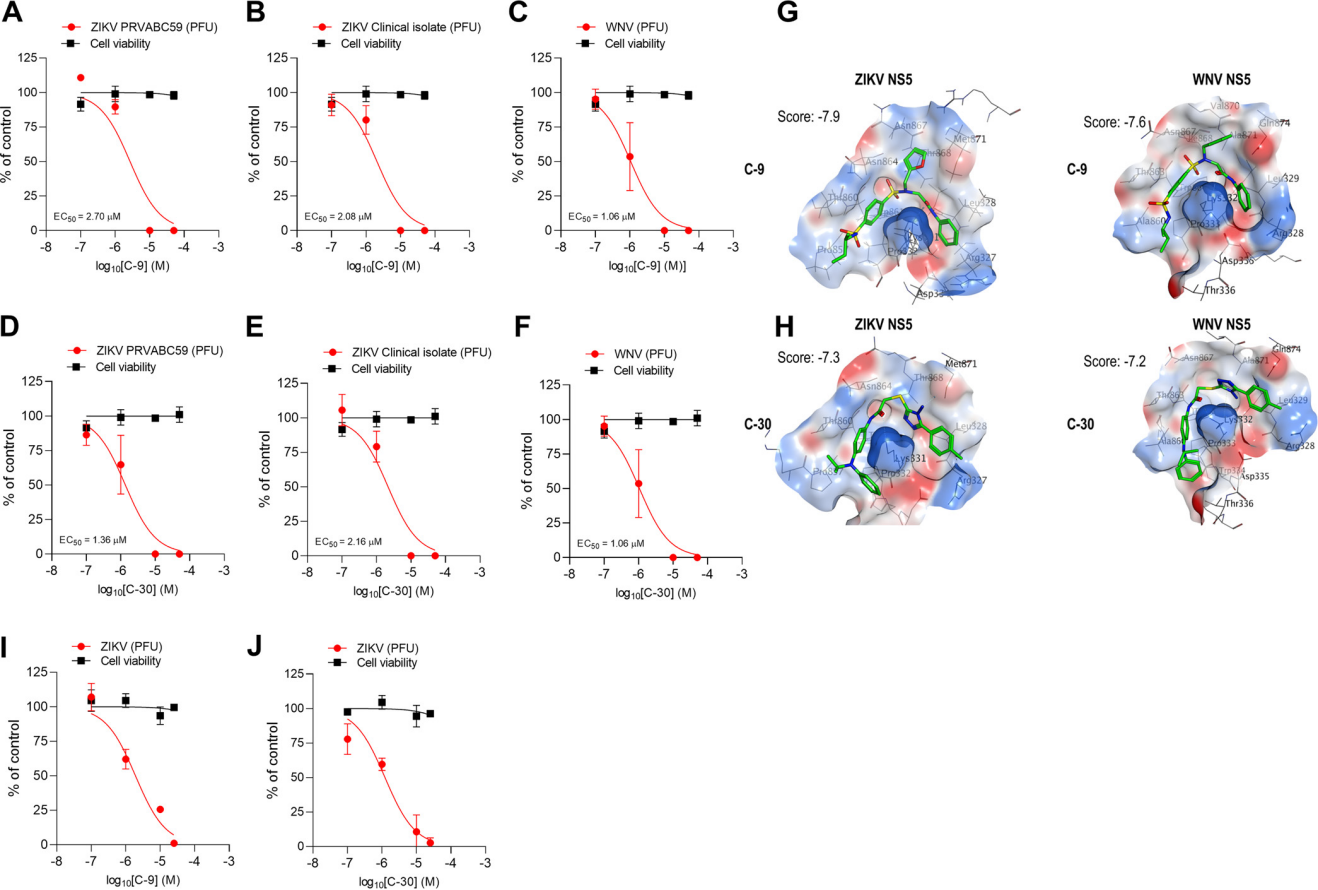
Broad-spectrum antiflaviviral profile of hit compounds C-9 and C-30. (A to C) Concentration-dependent inhibition of the replication of ZIKV (PVRABC59), ZIKV (clinical isolate), and WNV (NY99) in Vero cells treated with different concentrations of C-9 as determined by PRA. (D to F) Concentration-dependent inhibition of the replication of ZIKV, ZIKV (clinical isolate), and WNV in Vero cells treated with different concentrations of C-30 as determined by PRA. Graphs represent the mean ± SD of *n* = 3 independent experiments in duplicate. (G) Molecular basis of the interaction of C-9 with ZIKV and WNV NS5. Best pose for C-9 in ZIKV and WNV NS5. (H) Molecular basis of the interaction of C-30 with ZIKV and WNV NS5. Best pose for C-30 in ZIKV and WNV NS5. In panels G and H, selected residues are reported in sticks mode, and the final score (MM-GB/VI) after the flexible refinement is reported for each pose. (I) Concentration-dependent inhibition of ZIKV replication in human neural stem cells (hNSCs) treated with different concentrations of C-9 or 0.1% DMSO as a control. A curve representing hNSCs viability after 72 h at the same concentrations of C-9 relative to uninfected DMSO-treated hNSCs (considered 100% of cell viability) is also shown. (J) Concentration-dependent inhibition of ZIKV replication in hNSCs treated with different concentrations of C-30 or 0.1% DMSO as a control. A curve representing hNSCs viability after 72 h at the same concentrations of C-30 relative to uninfected DMSO-treated hNSCs (considered 100% of cell viability) is also shown. Graphs represent the mean ± SD of *n* = 3 independent experiments in duplicate.

Since the hit compounds were screened *in silico* searching for molecules able to interfere with the NS3-NS5 interaction of different flaviviruses (Fig. 1A), we next tested the activity against ZIKV of all of the nontoxic hits that showed an inhibitory effect either on DENV replication or on NS3-NS5 binding *in vitro* (Table S1). The EC_50_ values obtained for C-9 and C-30 against ZIKV were comparable to those obtained with DENV-2, while C-6 and C-24 were more active against ZIKV than DENV-2 (Fig. 4A and D; see also Table S3A in the supplemental material). We repeated the antiviral assays with a ZIKV clinical isolate and confirmed that both C-9 and C-30 inhibited different ZIKV strains comparably to the tested DENV serotypes (Fig. 4B and E; Table 1).

10.1128/mbio.03097-22.7 TABLE S3 Antiviral activity against different single-stranded positive RNA viruses. (A) Antiviral activity of selected hit compounds against ZIKV and WNV. (B) Antiviral activity of C-9, C-30, and reference compound NITD-008 against different single-stranded positive RNA viruses. Download Table S3, DOCX file, 0.01 MB. Copyright © 2023 Celegato et al. 2023 Celegato et al. https://creativecommons.org/licenses/by/4.0/ This content is distributed under the terms of the Creative Commons Attribution 4.0 International license.

We next tested some of the hit compounds active against ZIKV and DENV-2 against WNV (see Table S3A in the supplemental material). From these studies, it resulted that C-9, C-24, and C-30 also inhibited WNV replication in infected cells with an SI similar to that obtained for ZIKV (Fig. 4C and F; see also Table S3A). Thus, our results indicated that C-9 and C-30 have pan-dengue antiviral activity and broad-spectrum antiflaviviral potential. Interestingly, hit compound C-3 was active only against ZIKV. Again, by analyzing the best docking poses for C-9 and C-30 on NS5 of ZIKV and WNV, interactions of both compounds with the conserved K330 residue in cavity B were predicted (Fig. 1A and Fig. 4G and H). According to the predicted interaction with DENV NS5, other residues potentially involved in the interaction of C-9 and C-30 with NS5 of ZIKV and WNV are L327 and W859, which are conserved in all NS5 sequences of the considered flaviviruses (Fig. 1B). In WNV NS5, the predicted pose of C-9 slightly differs from DENV and ZIKV NS5 by presenting a switch in the orientation of the furan and phenyl rings. However, this switch does not affect the overall ligand conformation. Moreover, the predicted pose of C-30 retraced the U-shape conformation reported for DENV as well as for ZIKV and WNV. Residues A866 and Q869 of NS5 are conserved in both all DENV serotypes and WNV NS5, while in ZIKV, the glutamine is replaced by a methionine (M871) (Fig. 1A). This difference might explain the specific anti-ZIKV activity of C-3. Indeed, the hydrophobic ethyl-substituent in C-3 is oriented toward M871, guaranteeing further favorable contacts in comparison to the polar glutamine present in all DENV serotypes and WNV. Altogether, the predicted binding modes are in keeping with the broad-spectrum antiflaviviral activity that we observed for both hit compounds C-9 and C-30.

To better compare the activity of hit compounds C-9 and C-30 with a reference compound endowed with pan-flaviviral activity, we repeated the antiviral assays with NITD-008, an adenosine analog that selectively inhibits flaviviral RdRP. We observed that the EC_50_ values obtained for the hit compounds were in the same range of those for NITD-008 (except for ZIKV, for which NITD-008 was more potent) as reported in Table S3B. Given the different mechanism of action, these compounds could be evaluated for combination therapy.

To further assess the specificity of C-9 and C-30, we tested them against different positive-stranded RNA viruses, i.e., the alphavirus chikungunya virus (CHIKV) and a member of the *Picornaviridae* family, i.e., coxsackie virus B1 (COXVB1). Both compounds did not inhibit the replication of other unrelated positive-stranded RNA viruses (Table S3B); thus, we concluded that their antiviral activity was selective for the mosquito-borne flaviviruses that we initially considered (Fig. 1).

### C-9 and C-30 inhibit ZIKV replication in human neural stem cells.

ZIKV is known to infect fetal cells in the developing brain and to cause microcephaly in the case of congenital infection. For this reason, we evaluated the effects of C-9 and C-30 on the replication of ZIKV in human induced pluripotent stem cell (iPSC)-derived neural stem cells (hNSCs), which are a target *in vivo* of the virus. We infected hNSCs with ZIKV and then treated them for 120 h with different concentrations of C-9 and C-30 or 0.1% DMSO as a control. At the end of incubation, viral titers were determined by plaque assays in Vero cells. As reported in Fig. 4I to J, both C-9 and C-30 were able to reduce ZIKV titers obtained from infected hNSCs in a concentration-dependent manner. In the absence of ZIKV infection, C-9 and C-30 did not show toxic effects at the same concentrations and up to 250 μM (Fig. 4I to J; data not shown). Thus, the anti-ZIKV activity for these compounds was also confirmed in disease-relevant human cells that are targeted *in vivo* during congenital infection.

### Antiflavivirus profile of C-9 analogs.

Unfortunately, we were not able to repurchase a sufficient amount of compound C-9 to perform animal studies. Thus, we decided to test commercially available analogs of C-9 selected by substructure of the 4-sulfamoylbenzenesulfonyl portion (C-31 to C-44) (Fig. S1B) in order to evaluate their activity against different flaviviruses and *in vitro* against DENV-2 NS3-NS5 interaction. Among these analogs of hit C-9, C-42 was 4-fold more potent than the parental compound in inhibiting NS3-NS5 interaction *in vitro* and demonstrated broad antiviral activity against DENV, ZIKV, and WNV (see Table S4 in the supplemental material). However, the cytotoxicity was slightly higher. C-33, although demonstrating broad antiflavivirus activity in the low micromolar range against DENV-2, ZIKV, and WNV, did not show activity in disrupting DENV-2 NS3-NS5 interaction *in vitro* (Table S3). Thus, for this compound, we envisage a different mechanism of action that will be investigated in the future.

10.1128/mbio.03097-22.8 TABLE S4 Biological evaluation of commercially available analogs of hit compound C-9. Download Table S4, DOCX file, 0.01 MB. Copyright © 2023 Celegato et al. 2023 Celegato et al. https://creativecommons.org/licenses/by/4.0/ This content is distributed under the terms of the Creative Commons Attribution 4.0 International license.

### Efficacy of C-30 *in vivo* in a mouse model of DENV infection.

**FIG 5:**
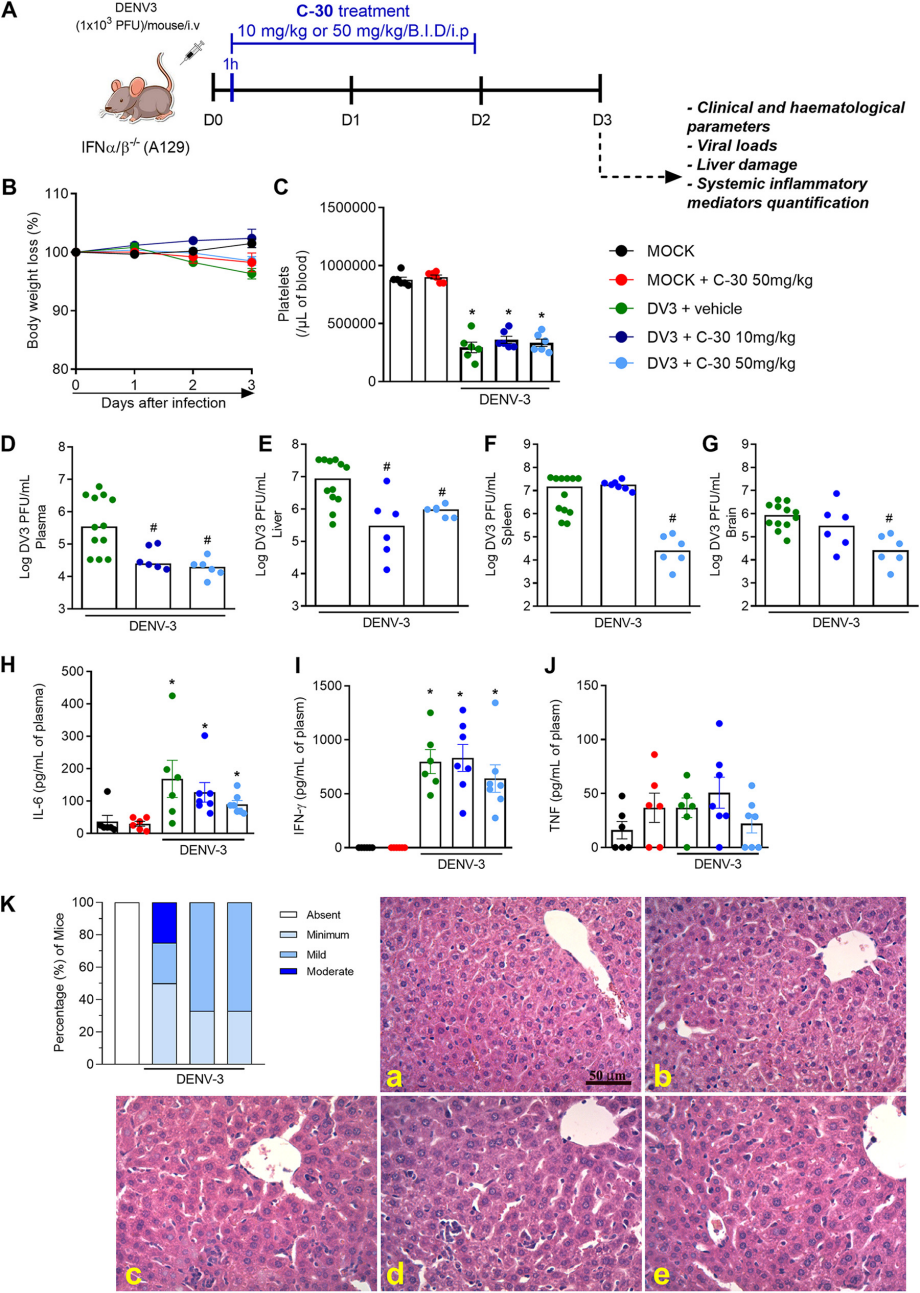
*In vivo* efficacy of C-30 in a mouse model of DENV-3 infection. (A) Experimental design, 8-week-old A129 mice were either mock infected or inoculated with 10^3^ PFU of DENV-3 by the intravenous (i.v.) route. From day 0 (1 h post-DENV-3 inoculation) and at 24 and 48 h, mice were treated or not twice a day with 5 mg/kg or 25 mg/kg of body weight of C-30 compound by the intraperitoneal (i.p.) route, b.i.d. (B) Body weight loss was assessed daily from day 0 until day 3 and expressed as a percentage of initial body weight. Mock (closed black circles), Mock treated with C-30 (closed red circles), DENV-3-infected mice treated with vehicle (green closed circles), or C-30 infected mice treated with either 10 mg/kg/day (dark blue closed circles) or 50 mg/kg/day (light blue closed circles; *n* = 6 to 12 mice/group). Differences over time and between treatments were compared by one-way ANOVA followed by Tukey’s multiple-comparison test. Three days after infection, animals were culled, and blood and tissues collected for the following analysis. (C) Platelet counts, shown as the number of platelets × 10^3^/μL of blood. Viral loads recovered from plasma (D), liver (E), spleen (F), and brain (G) of infected mice treated or not with C-30 examined by plaque assay in Vero cells. Results are shown as the log of PFU/mL of plasma or as the log of PFU/mg of spleen, liver, or brain (*n* = 6 to 12). All results are expressed as mean (horizontal bars). Analysis of cytokine production by quantification of IL-6 (H), IFN-γ (I), and TNF (J) in plasma of mock- and DENV-infected mice treated or not with C-30. Concentrations were assessed by ELISA and are shown as picograms per milliliter of plasma. In panels C to J, data were analyzed by one-way ANOVA followed by Tukey’s multiple-comparison test. *, *P* < 0.05 versus mock-infected group; #, *P* < 0.05 versus DENV-3-infected group. Histopathological analysis, liver of control and DENV-3-infected mice, treated or not with C-30, were collected, formalin-fixed, and processed into paraffin sections. (K) Histopathological scores of the inflammatory infiltrates concentrated in the perivascular area and (panels a to e) representative images of liver sections stained with H&E with spreading out into the liver parenchyma. Scale bar, 50 μm. (a) MOCK. (b) MOCK + C-30 (25 mg/kg b.i.d.). (c) DENV-3 + vehicle. (d) DENV-3 + C-30 (10 mg/kg/day). (e) DENV-3 + C-30 (50 mg/kg/day).

To validate further the NS3-NS5 dissociative strategy as a proof of concept *in vivo*, in the impossibility to test C-9, we decided to evaluate hit compound C-30 in a model of dengue infection using A129 mice (30). Compound C-30 was, therefore, subjected to resynthesis by the vendor Enamine; retested for structure, purity, and antiviral activity in cells; and finally evaluated in animal studies. The certificate of analysis of C-30 including the ^1^H spectrum, and mass spectrometry analysis can be found in the Text S1 in the supplemental material. In this study, male A129 mice were infected with 10^3^ PFU of a clinical isolate of DENV-3 (strain Eden-863DK1), and clinical parameters (body weight loss, circulating platelet counts, viral loads, liver damage, and production of systemic inflammatory mediators in the plasma) were analyzed at day 3 post-DENV-3 inoculation (Fig. 5A). C-30 treatment was started 1 h after DENV-3 inoculation, and the treatment was also repeated at 24 and 48 h postinfection. Treatment was performed twice a day (b.i.d.) at a final dose of 10 or 50 mg/kg of body weight per day per mouse. DENV-3 infection resulted in mild body weight loss (~5 to 8%) in the infected (DENV-3) and vehicle-treated groups compared to that of the mock-infected group and mice treated with C-30 or untreated mice (Fig. 5B). Similar body weight loss was observed in C-30-treated and DENV-3-infected mice when compared to that of vehicle-infected littermates (Fig. 5B). Thrombocytopenia, a clinical hallmark of DENV infection in humans (31), was observed in all infected groups with no difference between them (Fig. 5C). DENV-3 infection resulted in elevated viral loads in serum (Fig. 5D), spleen (Fig. 5E), liver (Fig. 5F), and brain (Fig. 5G) as observed in the vehicle-treated control group and consistent with previous reports (30). Treatment with C-30 resulted in a significant reduction of DENV-3 in plasma (Fig. 5D) and liver (Fig. 5E) when administered at both doses. Moreover, the 50 mg/kg dose of body weight of C-30 also significantly diminished viral loads in the spleen and in the brain (Fig. 5F and G, light blue symbols). These results demonstrate that C-30 exerts significant antiviral activity *in vivo* against DENV infection and is effective in reducing the virus replication in target tissues, including the brain. Next, several parameters were evaluated to determine the effects of C-30 in mitigating DENV-induced disease and inflammation. DENV-3 infection in A129 mice resulted in increased systemic levels of the proinflammatory mediators interleukin-6 (IL-6) (Fig. 5H) and gamma interferon (IFN-γ) (Fig. 5I) but not tumor necrosis factor (TNF) (Fig. 5J). C-30 treatment, regardless of the administered dose, had no effect on plasma levels of these mediators (Fig. 5H to J). Finally, hepatic damage induced by DENV-3 infection was assessed by histopathological analysis (Fig. 5K). In the vehicle-treated group, hepatic lesions were confirmed by the presence of inflammatory infiltrates concentrated in the perivascular area and spreading out into the liver parenchyma (Fig. 5K). The infiltrates were composed predominantly of mononuclear and polymorphonuclear cells, including neutrophils, macrophages, and multinucleated giant cells. C-30 treatment of DENV-3-infected mice (either at a dose of 10 or 50 mg/kg of body weight) was associated with reductions in histopathological inflammatory cell score damage as reported in Fig. 5K (panels d and e).

10.1128/mbio.03097-22.10 TEXT S1 Certificate of analysis: ^1^H spectrum and high-performance liquid chromatography mass spectrometry (HPLC-MS) analysis of compound C-30 (Enamine). Download Text S1, PDF file, 0.9 MB. Copyright © 2023 Celegato et al. 2023 Celegato et al. https://creativecommons.org/licenses/by/4.0/ This content is distributed under the terms of the Creative Commons Attribution 4.0 International license.

## DISCUSSION

Mosquito-borne flaviviruses threaten about half of the human population worldwide, but despite this, effective therapeutic agents are still lacking for most of these human pathogens. To test the hypothesis that targeting the highly conserved NS3-NS5 interface would lead to new small molecules with extended antiflaviviral activity and effectiveness *in vivo*, we applied a computational pipeline to virtually screen ~1 million small molecules aimed at identifying potential protein-protein interaction inhibitors able to bind NS5 and interfere with its interaction with NS3. Hit compounds C-9 and C-30 were selected for follow-up studies, given their favorable SI and their ability to specifically interfere with NS3-NS5 interaction in a concentration-dependent manner. In at-risk areas, often, coinfections with different flaviviruses can occur and reinfections with a different DENV serotype can lead to severe dengue; thus, the pan-serotype and broad-spectrum activity is an important feature when developing an effective antiflaviviral drug. By selecting a highly conserved target, our rationale was indeed to identify small molecules with therapeutic potential able to inhibit all four DENV serotypes and possibly other mosquito-borne flaviviruses. On this line, C-9 and C-30 exhibited similar efficacy against all four DENV serotypes in cellular assays. Computer-aided analysis of the binding mode proposed by molecular docking and NMR studies supported the binding at NS5 cavity B and pointed out residues that are conserved across all DENV serotypes and make crucial contacts with NS3 residues at the putative NS3-NS5 binding interface. The antiviral spectrum of hit compounds also extended to other relevant mosquito-borne flaviviruses lacking specific treatments, i.e., ZIKV and WNV, thus, further validating both the approach based on supervised molecular dynamics simulation (MSD) and the therapeutic potential of the hits.

The development of an effective pan-flaviviral antiviral drug might be challenging due to the differences in the sequences of viral proteins across family members. Moreover, even if the target is conserved, it has been demonstrated that mutations that confer drug resistance *in vitro* can be found in circulating strains, as in the case of NITD-688 (a candidate anti-DENV drug under late preclinical development that targets NS4B and possesses pan-serotype activity [17]), thus implicating the need for surveillance. DENV viremia is very short (~1 week), and hence, the emergence of drug-resistant viruses might be unlikely, while ZIKV was persistently found in body fluids of infected subjects for months (32). However, the current lack of approved, effective antiflaviviral drugs hampers the collection of clinical data regarding possible emergence of resistance *in vivo*. Our target-based approach, along with the selection of a flaviviral cold-spot for mutations, might ensure the low likelihood of emergence of escape mutants resistant to NS3-NS5 inhibitors. Indeed, under our experimental conditions, we were not able to isolate viruses with altered susceptibility to either C-9 or C-30. Data coming from ours and other groups obtained with protein-protein interaction inhibitors of influenza virus RNA polymerase PA-PB1 subunits and of DENV NS3-NS4B proteins indicated that compounds acting by disruption of PPI may have a high barrier to resistance (29, 33, 34). This seems to be confirmed also by the data obtained with NS3-NS5 inhibitors within this study. Noteworthy, since compounds belonging to this class act by a nonenzymatic mechanism, they could be associated with other antiflaviviral agents, e.g., enzymatic inhibitors, to prevent the emergence of resistant mutants and develop combination therapeutic strategies that could protect from infections by different flaviviruses.

Disruption of PPI relevant for virus replication has proven to be a very successful antiviral strategy (23, 35); however, this approach was, until recently, poorly explored in antiflaviviral drug discovery. Most of the existing work had been focused on the identification of inhibitors of the NS2B/NS3 protease complex of DENV and ZIKV (36–41). Recently, JNJ-A07, a compound with potent pan-dengue antiviral activity and the ability to block the interaction between NS3 and NS4B of DENV, was reported by Kaptein and colleagues (34). This compound holds promising features for the development of anti-DENV drugs acting by a nonenzymatic mechanism, but unfortunately it is not active against other flaviviruses. Only a few molecules with multiple mechanisms and targets were reported to interfere with DENV NS3-NS5 interaction (42–44). We applied, for the first time, molecular dynamics simulation and a rational structure-guided screening to identify small-molecule NS3-NS5 inhibitors and demonstrated their broad antiviral activity against different flaviviruses, highlighting the therapeutic potential of an approach based on the disruption of a conserved protein-protein interaction. Moreover, for the first time, we demonstrated that an NS3-NS5 inhibitor is effective *in vivo* in a mouse model of DENV infection, as it was able to reduce viral replication in different target organs and to ameliorate virus-induced pathology.

This study has some limitations. First, these small molecules represent a proof of concept and need further optimization aimed at improving the solubility and pharmacological profile and at reducing the effective dose *in vivo*. Nevertheless, they represent new chemical scaffolds that could be further developed in antiflavivirus drugs with extended spectrum. We provided evidence of the pan-dengue activity and broad antiflaviviral potential of the small molecules in the cellular context, but we only demonstrated the *in vivo* efficacy of C-30 in the animal model of DENV-3 infection. Considering the high conservation of the target across flaviviruses, we expect that these compounds might be active *in vivo* against ZIKV and WNV also, as was also shown in infected cells. In future studies with optimized NS3-NS5 inhibitors, the efficacy should be tested *in vivo* against all DENV serotypes and other mosquito-borne flaviviruses.

In conclusion, our study represents a seminal step forward in the development of much awaited broad-spectrum antiflaviviral agents that could be used, alone or in combination with other drugs targeting viral enzymatic functions, for the treatment of the infections associated with different mosquito-borne flaviviruses.

## MATERIALS AND METHODS

### Computational studies.

A library of ~1 million commercially available compounds from Asinex (http://www.asinex.com), Enamine (https://enamine.net), and Life Chemicals (https://lifechemicals.com/) was prepared for VS using OpenEye OEChem toolkit (OpenEye Scientific Software Inc. [2020]; OEChem, [http://www.eyesopen.com]) and LigPrep of Schrodinger suite (Schrödinger Release 2020-4; Maestro, Schrödinger, LLC, New York, NY) to obtain a good starting conformation, a suitable ionic and tautomeric state according to our ligand preparation pipeline MMsINC (45). Compounds containing PAINS moieties were filtered out with RDkit (RDKit; Open-source cheminformatics, https://www.rdkit.org).

Coordinates of the DENV NS5 protein were retrieved from the Protein Data Bank (PDB) (ID number 5k5m, chain A) (12). The structure was then prepared for the VS using the Structure Preparation tool part of the Molecular Operating Environment 2018.01 suite (MOE) (Chemical Computing Group [CCG] Inc.; Molecular Operating Environment [MOE], [http://www.chemcomp.com]). The correct protonation state for titratable residues was set using the Protonate3D tool (pH 7, T 310 K, salt 0.154). Hydrogen atoms were further energy minimized using AMBER10 (46) force field until a gradient of 0.1 Kcal mol-1 Å-2 was reached. Finally, all water molecules, ligands, and buffer components were removed. For the VS, the Protein-Ligand ANT System (PLANTS) molecular docking engine was used coupled with the ChemPLP scoring function (47). For each compound, 10 poses were generated using an RMSD threshold of 1.5 Å to remove similar ligand conformation. The resulting poses were filtered considering the top-scoring ligands and removing the poses with the positive van der Waals and electrostatic interaction energy (calculated with MOE). The highest scoring pose for each ligand was retained, and the 3,000 best scoring ligands were promoted to a further rescoring procedure according to the MM-GBSA scheme (Prime tool; Schrodinger 2020-04; Maestro, Schrödinger, LLC, New York, NY). For the docking calculations on ZIKV and WNV, their NS5 3D conformations were respectively retrieved from the Protein Data Bank (https://www.rcsb.org/) (ID number 6LD1 and 2HCN) X-ray structures (48, 49) and prepared similarly to DENV NS5. The docking calculation of the VS hits on ZIKV and WNV NS5 was carried out with the previously described protocol. C-9 and C-30 poses on NS5 from DENV, ZIKV, and WNV were further refined by the refining procedure implemented in the Docking tool of MOE to consider also the flexibility of the protein side chain and scored with MM-GBVI procedure.

### Compounds, cells, and viruses.

Compounds 1 to 9 and 31 to 44 were purchased from Asinex (The Netherlands), while compounds 10 to 30 were purchased from Enamine (Estonia). Compound Flu-1 was previously described (23). Reference compound NITD-008 was purchased from Merck. All compounds were dissolved in DMSO, and stock solutions were stored at −20°C.

African green monkey Vero cells from ATCC (VR-CCL-81) were cultured in Dulbecco modified Eagle medium (DMEM) supplemented with 10% fetal bovine serum (FBS) (Life Technologies) in the presence of 100 U/mL penicillin and 100 μg/mL streptomycin (Life Technologies) and were maintained at 37°C in a humidified atmosphere supplemented with 5% CO_2_. Human neural stem cells (hNSCs) were derived from UNIPDi007, a human induced pluripotent stem cell (hiPSCs), as previously described (50). Briefly, hiPSCs growing on Matrigel (Corning) with mTeSR-1 (StemCell Technologies) medium were detached by Accutase (StemCell Technologies) treatment and seeded in 6-well Matrigel-coated plates in mTeSR-1 at a density of 2.5 × 10^5^ cells/well. The next day, a medium composed by Neurobasal and Neural induction supplement (both from Gibco) was added to the cells and was changed every other day from day 0 to day 7. Starting from day 7, hNSCs were passaged with Accutase in NSCs-expansion medium (NEM) composed by 50% of Neurobasal medium, 50% of Advanced DMEM/F12 (Gibco), and neural induction supplement. Rock Inhibitor (RI) (StemMACS) to a final concentration of 5 μM was added at every passage. hNSCs were characterized by seeding them on Matrigel-coated coverslips and by immunostaining with anti-SOX2 (GeneTex) and anti-Nestin (Abcam) antibodies. Vero CCL81 (code 0245) and Aedes albopictus C6/36 (code 0343) cells were obtained from the Banco de Células do Rio de Janeiro (BCRJ) repository and cultured in RPMI 1640 medium (CultiLab) or L15 medium (CultiLab), respectively, supplemented with 10% of inactivated fetal bovine serum (CultiLab). All cell lines were routinely tested for mycoplasma contamination using a quantitative PCR (qPCR)-based in-house developed assay.

DENV-1 (TH-Sman strain) and DENV-2 (TH-36 strain) were purchased from ATCC. DENV-2 (NGC strain), DENV-3 (H-87 strain), DENV-4 (H-241 strain), Zika virus (PRVABC59 strain), and WNV (NY99 strain) were purchased from the National Collection of Pathogenic Viruses (NCPV) (Public Health England, UK). ZIKV, CHIKV, and COXVB1 were clinical strains isolated at Padua University Hospital (Padua, Italy). All viruses were propagated and titrated in Vero cells. All work with infectious DENV and WNV was performed in a biosafety level 3 (BSL3) laboratory according to the safety practices as approved by the Department of Molecular Medicine (University of Padua, Italy) Committee on Microbiological Safety. For *in vivo* experiments, low passage human clinical isolates of DENV-3 (EDEN 863) was propagated in Aedes albopictus C6/36 cells. All *in vivo* studies with the infectious viruses were performed in a BSL-2 facility of the Immunopharmacology lab of the Institute of Biological Sciences at Universidade Federal de Minas Gerais (UFMG).

### Antiviral assays.

For plaque reduction assays (PRAs) with the different flaviviruses (DENV-2, ZIKV, WNV), Vero cells were seeded at a density of 4 × 10^5^ cells per well in 12-well plates. The next day, the cells were first washed with serum-free medium and then infected at 37°C with 30 PFU per well of the different viruses in serum-free DMEM, and plates were rocked every 15 min. At the end of incubation, the inoculum was removed, and medium containing various concentrations of test compounds, 2% FBS, and 1.2% Avicel microcrystalline cellulose (FMC BioPolymer) was added. After an incubation period that depended on the virus (7 days for DENV-2 and 3 days for ZIKV and WNV), cell monolayers were fixed and stained and plaques were counted. For PRAs with CHIKV and COXVB1, Vero cells were seeded at a density of 1 × 10^4^ cells per well in 24-well plates. The next day, the cells were first washed with serum-free medium and then infected at 37°C with 80 PFU per well of the different viruses in serum-free DMEM. At the end of incubation, the inoculum was removed, and medium containing various concentrations of test compounds, 2% FBS, and 0.6% methylcellulose (Merck) was added. After an incubation period that depended on the virus (2 days for CHIKV and 3 days for COXVB1), cell monolayers were fixed and stained, and plaques were counted.

For viral RNA reduction assays with DENV serotypes 1 to 4, Vero cells were seeded at a density of 1 × 10^5^ cells per well in 24-well plates. The next day, the cells were infected at 37°C with the different viruses in serum-free DMEM at a multiplicity of infection (MOI) of 0.001, and plates were rocked every 15 min. At the end of incubation, the inoculum was removed and medium with 2% FBS containing various concentrations of test compounds was added. Viral RNA was isolated from the culture supernatant at 72 hours postinfection (h p.i.), using the PureLink Viral RNA/DNA minikit (Invitrogen) according to the manufacturers’ instructions. Viral RNA was quantified using the one-step reverse transcriptase PCR (RT-PCR) kit dengue virus subtypes 1, 2, 3, and 4 PCRmax (Cole Parmer, UK), according to the manufacturers’ instructions, on a 7000 real-time PCR platform (Applied Biosystems). Effective concentration at half-maximal response (EC_50_), obtained by either plaque assays or reverse transcriptase quantitative PCR (RT-qPCR), were determined by analysis with nonlinear regression function of GraphPad Prism 8.0.

For antiviral assays in human NSCs (hNSCs), cells were plated at 8 × 10^3^ cells per well in 96-well plates, incubated overnight, and infected the next day with ZIKV at an MOI of 1 PFU/cell. After virus adsorption for 2 h at 37°C, cells were incubated with 0.2 mL of fresh NEM in the absence or in the presence of different concentrations of test compounds. Plates were incubated for 120 h at 37°C and then subjected to one cycle of freezing and thawing. ZIKV titers were determined by transferring 0.02-mL aliquots from each hNSCs well (followed by 1:10 serial dilutions across the plate up to 10^−7^) to a fresh 12-well monolayer culture of Vero cells. Cultures were incubated for 72 h, and at the end of incubation, they were fixed and stained and the numbers of plaques were determined.

### Cell viability assays.

The effects of test compounds on cell viability were assessed by the 3-(4,5-dimethylthiazol-2-yl)-2,5-diphenyl tetrazolium bromide (MTT) method at 72 h posttreatment as previously described (51).

### Expression and purification of DENV-2 NS5-RdRP.

The pET28a^+^-DEN2-Pol plasmid containing the NS5 RNA-dependent RNA polymerase (RdRP) domain (sequence from residue 272 to residue 900 of DENV-2 NGC) (GenBank accession number KM204118.1) was obtained by PCR amplification using primers D2_NS5(262-900) FOR and REV (see Table S2B in the supplemental material) and cloning into pET28a^+^ plasmid (Merck) at BamHI/SalI sites. The fusion construct was verified by sequencing. To obtain the 6×His-NS5(272-900) protein, a procedure described in reference 52 was followed with modifications. pET28a^+^-DEN2-Pol plasmid was transformed into Escherichia coli strain BL21(DE3)pLysS (StrataGene). Three liters of cells were grown in Luria Bertani (LB) medium containing 100 μg/mL ampicillin until the optical density at 600 nm (OD_600_) was 0.5. Then, cells were cooled at 16°C, and protein expression was induced by the addition of 0.5 mM isopropyl-β-d-thiogalactopyranoside (IPTG, ICN) overnight (O/N) at 16°C. Cells were pelleted, resuspended in resuspension buffer (20 mM Tris-HCl pH 8.0, 500 mM NaCl, 10 mM β-mercaptoethanol, 10% glycerol) added with 1 mg/mL lysozyme and complete protease inhibitors (Roche Molecular Biochemicals), and then lysed by two freeze/thaw cycles and by sonication. The lysate was centrifuged at 16,000 × *g* for 30 min, applied to a 0.5 mL-Ni-nitrilotriacetic acid (Ni-NTA) agarose resin column (Qiagen) that had been equilibrated in resuspension buffer. The column was washed with resuspension buffer added with 15 mM imidazole, and then protein was eluted with 20 mM Tris-HCl pH 8.0, 500 mM NaCl, 10 mM β-mercaptoethanol, and 125 mM imidazole.

### Expression and purification of DENV-2 NS3-Hel.

The pRSET-DEN2-NS3-Hel plasmid containing the NS3 helicase domain (sequence from residue 177 to residue 618 of DENV-2 NGC) was obtained by PCR amplification using primers D2_NS3(177-618) FOR and REV (Table S2B) and cloning into the pRSETA plasmid (Thermo Fisher) at BamHI/HindIII sites. The fusion construct was verified by sequencing. To obtain the 6×His-NS3(177-618) protein, the pRSET-DEN2-NS3-Hel plasmid was transformed into E. coli strain BL21(DE3)pLysS (StrataGene). Three liters of cells were grown in LB medium containing 100 μg/mL ampicillin until the OD_600_ was 0.7. Then, cells were cooled at 16°C, and protein expression was induced by the addition of 0.4 mM IPTG O/N at 16°C. Cells were pelleted, resuspended in resuspension buffer (20 mM Tris-HCl pH 8.0, 500 mM NaCl, 0.5 M urea, 25 mM imidazole) added with 1 mg/mL lysozyme and complete protease inhibitors (Roche Molecular Biochemicals), and then lysed by two freeze/thaw cycles and by sonication. The lysate was centrifuged at 16,000 × *g* for 30 min and applied to a 0.5-mL Ni-NTA agarose resin column (Qiagen) that had been equilibrated in resuspension buffer. The column was washed with resuspension buffer added with 25 mM imidazole, and then the protein was eluted with 20 mM Tris-HCl pH 8.0, 500 mM NaCl, 10 mM β-mercaptoethanol, and 125 mM imidazole. Both 6×His-NS5(272-900) and 6×His-NS3(177-618) purified proteins were dialyzed against 20 mM Tris-HCl (pH 8.0), 150 mM NaCl, 30% (vol/vol) glycerol, and 5 mM dithiothreitol (DTT) and stored at −80°C.

### DENV-2 NS3-NS5 interaction ELISA-based assay.

Microtiter plates (96-well; Nuova Aptaca) were coated with 300 ng of purified 6×His-NS3(177-618) for 3 h at 37°C and then blocked with 2% (wt/vol) bovine serum albumin (BSA) (Sigma) in phosphate buffer saline (PBS) for 2 h. After washes with PBS containing 0.3% Tween 20, 200 ng of 6×His-NS5(272-900) was added and incubated O/N at 20°C in the absence or the presence of test compounds. After washing with PBS-0.3% Tween 20, samples were then incubated with anti-NS5 antibody (Thermo Fisher; diluted 1:3,000 in PBS containing 2% FBS) for 2 h at room temperature (RT) and successively with horseradish peroxidase (HRP)-conjugated goat anti-mouse secondary antibody (Millipore; diluted 1:5,000 in PBS containing 2% FBS) for 2 h at RT. Following washes with PBS-0.3% Tween 20, the chromogenic substrate 3,3′,5,5′ tetramethylbenzidine (TMB) (KPL Inc.) was added and acidified with 3.6% HCl. Absorbances were read at 450 nm using a spectrophotometer microplate reader (Multiskan FC; Thermo Fisher Scientific). To test the specificity of the assay but employing 6×His-IE2(290-579) protein, a specific antibody recognizing human cytomegalovirus IE2 was used in the ELISA. The 6×His-IE2(290-579) protein was previously described (53). 6×His-PA(239-716) and GST-PB1(1-25) proteins and the ELISA-based assay for the interaction of influenza A virus PA and PB1 proteins were also described elsewhere (23).

### Protein expression analysis.

For Western blot analysis, Vero cells in 12-well plates were pretreated for 2 h with different concentrations of C-9, C-30, or 0.1% DMSO as a control and then infected with DENV-2 NGC at an MOI of 0.5 PFU/cell. Following virus adsorption, infected cells were incubated with different concentrations of C-9, C-30, or 0.1% DMSO as a control. Whole-cell protein extracts were prepared at 56 h p.i. and then analyzed by Western blotting with different antibodies listed in Table S2C. Immunocomplexes were detected with the appropriate secondary anti-immunoglobulin antibodies conjugated to horseradish peroxidase (Life Technologies).

For immunofluorescence analysis, Vero cells in 24-well plates on coverslips were pretreated for 2 h with different concentrations of C-9, C-30, or 0.1% DMSO as a control and then infected with DENV-2 NGC at an MOI of 0.5 PFU/cell. Following virus adsorption, infected cells were incubated with different concentrations of C-9, C-30, or 0.1% DMSO as a control. At 56 h p.i., cells were fixed with 4% paraformaldehyde in PBS 1× for 24 h and then permeabilized with 0.1% Triton X-100 in PBS 1× for 10 min at RT. After extensively washing with PBS, cells were incubated first with 4% FBS in PBS 1× for 1 h at RT and then with anti-Env protein primary antibody (listed in Table S2C) diluted in FBS 4% in PBS for 1 h at 37°C under shaking. Cells were then washed extensively with 4% FBS in PBS 1× and incubated with secondary fluorochrome-conjugated antibody (Table S2C) for 1 h at 37°C. Nuclei were stained by incubation for 20 min with Draq5 (1:8,000 in PBS 1×). Cells were imaged using a Nikon Eclipse Ti-E microscope.

### Binding studies by nuclear magnetic resonance.

The NMR experiments were acquired at 298 K on a Bruker 600 MHz spectrometer equipped with an autosampler (64 samples) and a nitrogen-cooled cryoprobe. C-9 and C-30 compounds were tested against 6×His-NS5(272-900) performing water-ligand observed via gradient spectroscopy (WaterLOGSY) experiments (54) in the presence and in the absence of the protein. The protein was buffer exchanged into PBS, and each NMR sample contained a 50 μM compound in 0.1% deuterated DMSO (C-30) or DMSO (C-9), 5% (vol/vol) deuterated water, and 0.5 μM 6×His-NS5. As an internal standard, 4,4-dimethyl-4-silapentane-1-sulfonic acid (DSS) was finally added to each NMR tube (50 μM). The WaterLOGSY experiments were acquired with 480 scans, a recovery delay of 4.5 s before each scan, and a mixing time of 1.5 s. They were performed with a 180° inversion pulse applied to the water signal at 4.7 ppm using a Gaussian-shaped selective pulse of 7.5 ms, and water suppression was achieved by the excitation sculpting pulse scheme (55). The NMR spectra were processed and analyzed by Topspin 4.3.1 (Bruker BioSpin GmbH, Rheinstetten, Germany).

### Isolation of DENV-2 viruses resistant to C-9 and C-30.

To evaluate the barrier to drug resistance of C-9 and C-30, we followed a procedure similar to that described for influenza virus (29). DENV-2 NGC was serially passaged in the presence of increasing concentrations of C-9, C-30, or 0.1% DMSO as a control. Confluent Vero cells in 24-well plates were infected with DENV-2 at an MOI of 0.1 PFU/cell and then treated with C-9 or C-30 or 0.1% DMSO as a control. Cell supernatants were harvested at 72 h p.i., and 0.1 mL was used to infect Vero cells in the presence of increasing amounts of test compounds. The concentrations of C-9 and C-30 applied to the first passage (P1) corresponded approximately to that of its EC_50_ value in PRAs against DENV-2 (Table 1); successively, compound concentration was systematically increased until the 4th passage (P5) and then kept constant (approximately the EC_90_) for 5 additional passages (Table S2A). At selected passages, the drug sensitivity of harvested viruses was determined by PRA as described above, using parental DENV-2 NGC virus as a control.

### Animal studies.

This study was carried out in accordance with the Brazilian Government’s ethical and animal experiments regulations (law 11794/2008). The experimental protocol was approved by the Institutional Animal Care and Use Committee of the UFMG (CEUA/UFMG, Brazil, permit protocol number 234/2019). All surgeries were performed under ketamine/xylazine anesthesia, and all efforts were made to minimize animal suffering. Male type I interferon receptor-deficient mice (A129), SV129 background, obtained from Bioterio de Matrizes da Universidade de Sao Paulo (USP, Brazil), were bred and maintained at animal facilities of the Immunopharmacology Laboratory of the UFMG. For experiments, 8-week-old A129 mice were kept under specific pathogen-free conditions at a constant temperature (25°C) with free access to chow and water in a 12-h light/dark cycle.

For DENV infection experiments, mice received an intravenous injection of 10^3^ PFU of the DENV-3 strain (EDEN 863) (30, 56). A129 mice were treated with C-30 (10 or 50 mg/kg/animal; intraperitoneally [i.p.], b.i.d.) from day 0 (1 h postinfection) and every 24 h until sacrifice (day 3). Mice were randomly allocated into experimental groups using an MS Excel randomization tool. All experiments were repeated twice. For platelets quantification, murine blood was obtained from the cava vein in heparin-containing syringes at the indicated time points under ketamine and xylazine anesthesia (100 mg/kg and 10 mg/kg of body weight, respectively). The final concentration of heparin was 50 U/mL. Platelets were counted in a Neubauer chamber (57). Results are presented as the number of platelets per microliter of blood. For cytokines quantification in plasma, the concentrations of murine IL-6, IFN-γ, and TNF in plasma samples were measured using commercially available DuoSet ELISA development kits (R&D). All of the immunoassays were performed according to manufacturer’s instructions. For virus titrations, A129 mice were assayed for viral titers in plasma, spleen, liver, and brain. Blood samples were collected in heparinized tubes and centrifuged at 3,000 × *g* for 15 min at RT. The plasma was collected and stored at −80°C until assayed. For virus recovery from the spleen, liver, and brain, the organs were collected aseptically at day 3 post-DENV-3 infection and stored at −80°C until assayed. Tissue samples were weighed and grounded using a pestle and mortar and prepared as 10% (wt/vol) homogenates in RMPI 1640 medium without fetal bovine serum (FBS). Viral loads in the supernatants of tissue homogenates and plasma samples were assessed by direct plaque assay using Vero CCL-81 cells as previously described (30). Results were measured as PFU per 100 mg of tissue weight or per mL of plasma. The limit of detection of the assay was 100 PFU/g of tissue or per mL. For histopathological analysis, liver samples from euthanized mice were obtained at day 3 upon DENV-3 inoculation. After that, samples were immediately fixed in 10% neutral buffered formalin for 24 h and embedded in paraffin. Tissue sections (4-μm thicknesses) were stained with hematoxylin and eosin (H&E) and evaluated under a microscope Axioskop 40 (Carl Zeiss, Göttingen, Germany) adapted to a digital camera (PowerShot A620; Canon, Tokyo, Japan). Histopathology score was performed as previously described (30), evaluating inflammatory cell infiltrate added to a five-point score (0, absent; 1, minimal; 2, slight; 3, moderate; 4, marked; and 5, severe) in each analysis. A total of two sections for each animal were examined, and results were plotted as a percentage of mice expressing each score.

### Statistical analysis.

All statistical analyses were performed using GraphPad Prism software version 8.0. The results were analyzed using appropriate statistical tests as indicated in figure legends.

### Data availability.

All study data are included in the article and/or in the supplemental material.
