# Supplementary material for: Small-Molecule Inhibitor of Flaviviral NS3-NS5 Interaction with Broad-Spectrum Activity and Efficacy In Vivo
Source: mBio. 2023 Jan 9;14(1):e03097-22. doi: 10.1128/mbio.03097-22 (PMC9973282; doi:10.1128/mbio.03097-22)
Supplement: FIG S1 [file mbio.03097-22-s0001.docx]

Figure S1A Chemical structures of the 30 hit compounds identified by virtual screening.

**
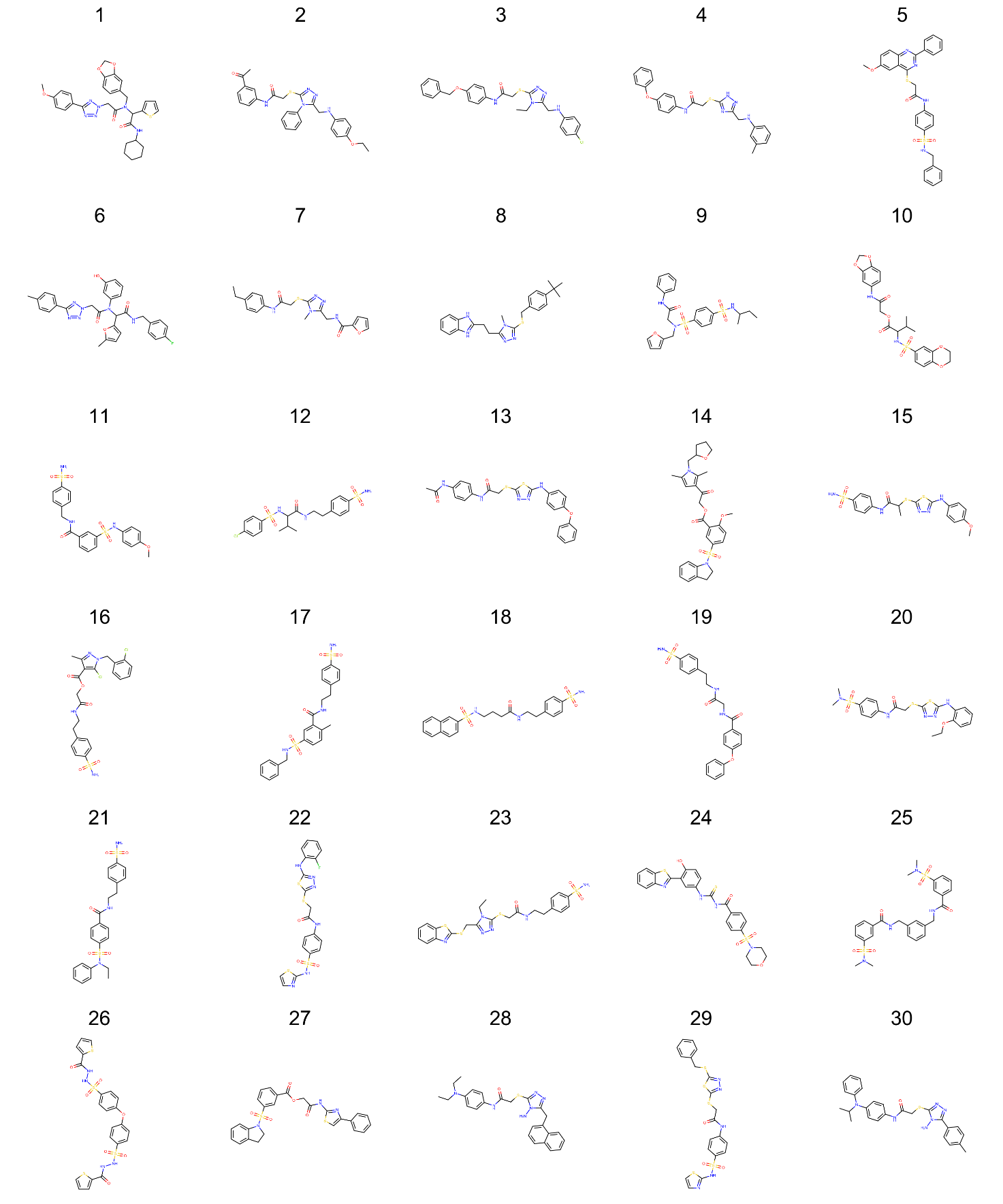
**

**Figure S1B** **Chemical structures of the analogs of hit compound C-9.**

**_
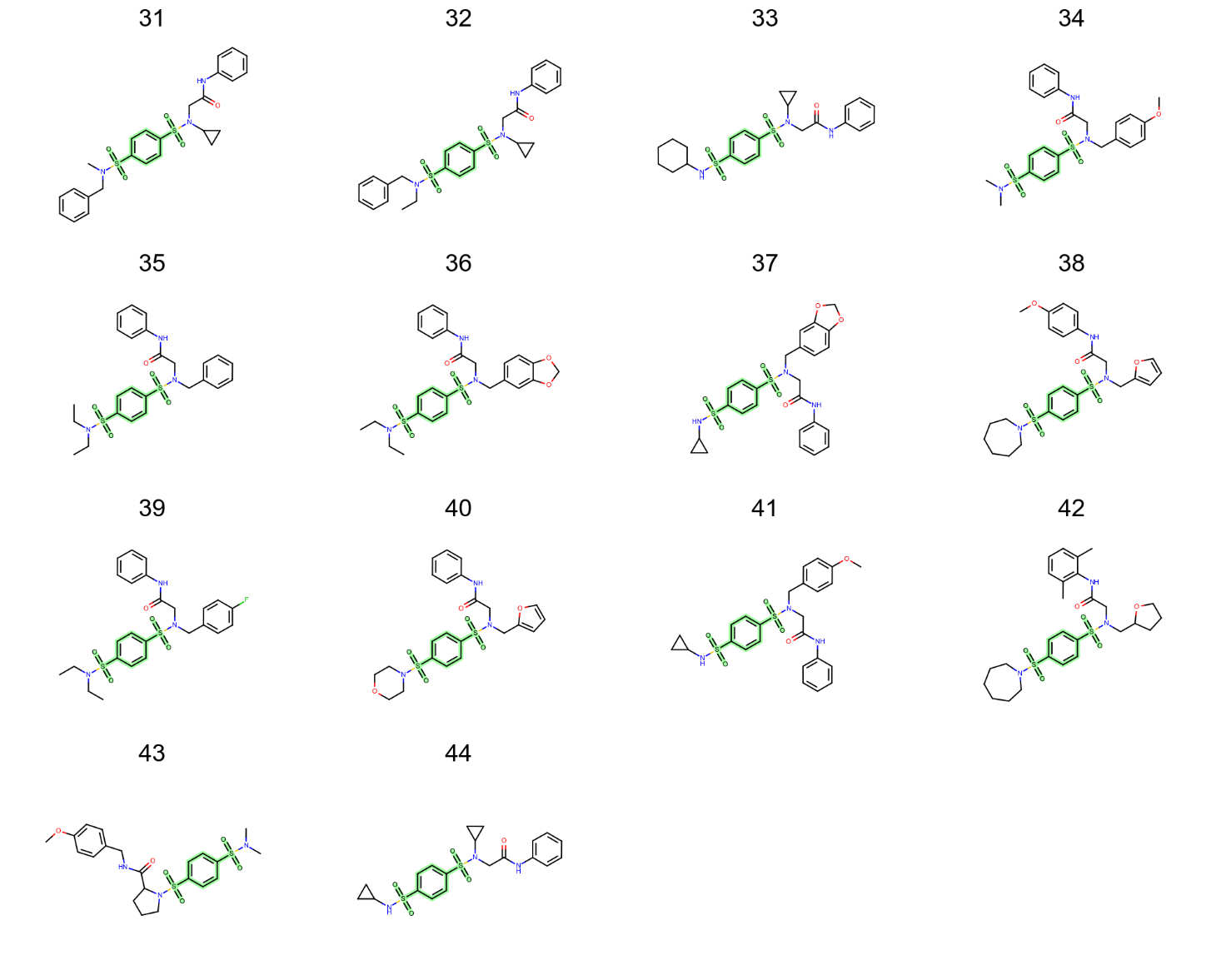
_**
