# Supplementary material for: Small-Molecule Inhibitor of Flaviviral NS3-NS5 Interaction with Broad-Spectrum Activity and Efficacy In Vivo
Source: mBio. 2023 Jan 9;14(1):e03097-22. doi: 10.1128/mbio.03097-22 (PMC9973282; doi:10.1128/mbio.03097-22)
Supplement: FIG S2 [file mbio.03097-22-s0002.docx]

**Figure S2. Concentration-dependent inhibition of DENV-2 NGC replication by selected hit compounds.** Plaque reduction assays were performed in Vero cells infected with DENV-2 NGC strain and treated with different doses (from 0.1 to 50 µM) of the indicated test compounds. Graph represents mean ± SD of n ≥ 3 independent experiments in duplicate. Reported are concentration-response curves for 9 out of 30 hit compounds.
