# Supplementary material for: Small-Molecule Inhibitor of Flaviviral NS3-NS5 Interaction with Broad-Spectrum Activity and Efficacy In Vivo
Source: mBio. 2023 Jan 9;14(1):e03097-22. doi: 10.1128/mbio.03097-22 (PMC9973282; doi:10.1128/mbio.03097-22)
Supplement: FIG S3 [file mbio.03097-22-s0003.docx]

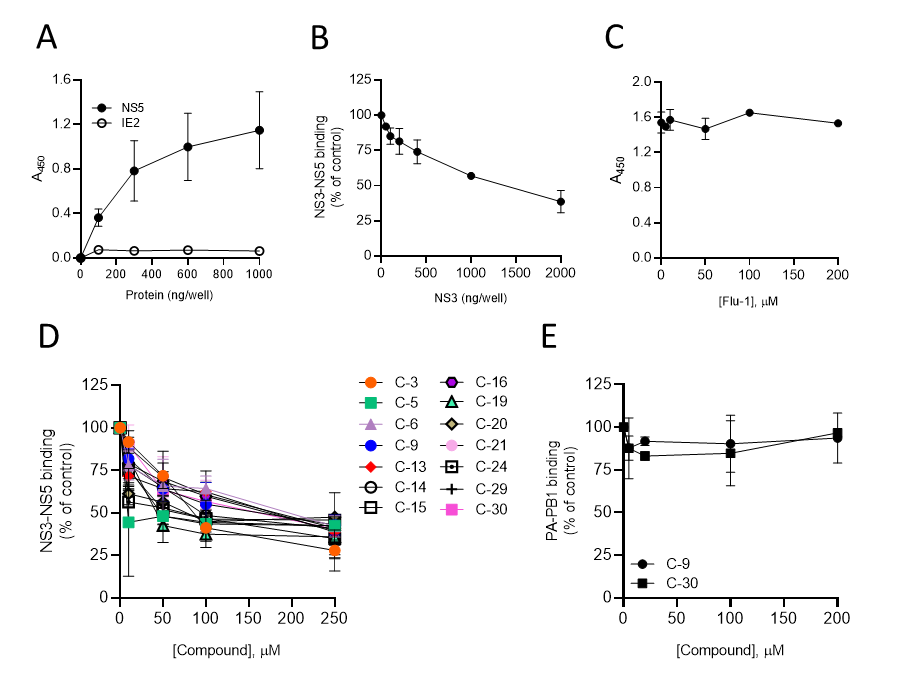


**Figure S3. DENV-2 ELISA-based NS3-NS5 interaction assay.** (A) Concentration-dependent increase in absorbance when increasing amounts of 6His-NS5(279-900), but not of unrelated 6His-IE2(290-579), were added to wells coated with 300 ng of 6His-NS3(177-618). (B) The addition to the ELISA NS3-NS5 mixture of increasing amounts of free 6His-NS3(177-618) resulted in a concentration-dependent reduction in absorbance due to competitive binding to unbound NS5. (C) The addition to the ELISA NS3-NS5 mixture of increasing amounts of Flu-1, a previously described dissociative inhibitor of influenza virus PA-PB1 interaction, did not affect NS3-NS5 binding. Data shown represent the mean ± SD of n = 3 independent experiments. (D) Concentration-dependent inhibition of NS3-NS5 interaction in ELISA-based assays *in vitro*. Increasing concentrations of the indicated compounds were added together with 200 ng of 6His-NS5(279-900) to wells coated with 300 ng of 6His-NS3(177-618). Binding of 6His-NS5(279-900) was quantified as described in Fig. S3. Data shown represent the mean ± SD of n = 3 independent experiments. Reported are dose-response curves for 14 out of 30 hit compounds. (E) Absence of inhibitory effects of C-9 and C-30 on influenza virus RNA polymerase subunits PA-PB1 interaction *in vitro*.
