# Supplementary material for: Small-Molecule Inhibitor of Flaviviral NS3-NS5 Interaction with Broad-Spectrum Activity and Efficacy In Vivo
Source: mBio. 2023 Jan 9;14(1):e03097-22. doi: 10.1128/mbio.03097-22 (PMC9973282; doi:10.1128/mbio.03097-22)
Supplement: TABLE S1 [file mbio.03097-22-s0005.docx]

Table S1 Antiviral activity and cytotoxicity of hit compounds against DENV-2

| **Hit #** | **ZINC CODE** | **Antiviral Activity**  **EC_50_^a^ (µM)** | **Cytotoxicity**  **CC_50_^b^ (µM)** | **SI^c^** | **NS3-NS5 ELISA**  **IC_50_^d^ (µM)** |
| --- | --- | --- | --- | --- | --- |
| **C-1** | 00888548 | 49.2 ± 7.2 | 100 ± 26 | 2 | >250 |
| **C-2** | 00649353 | >50 | >250 | >5 | >250 |
| **C-3** | 00649432 | >50 | 41.8 ± 1.7 | <1 | 83.2 ± 18.6 |
| **C-4** | 00873573 | 15.5 ± 7.7 | 112 ± 8 | 7 | 203.0 ± 60.0 |
| **C-5** | 00906272 | >10 | 5.2 ± 0.9 | <1 | 28.3 ± 16.6 |
| **C-6** | 00888078 | 32.5 ± 10.6 | 72.8 ± 28.3 | 2 | 170 ± 68 |
| **C-7** | 04816414 | >50 | >250 | >5 | >250 |
| **C-8** | 00929959 | 23.3 ± 3.7 | 36.8 ± 9.0 | 2 | >250 |
| **C-9** | **01333392** | **2.4 ± 1.3** | **>250** | **>104** | **161 ± 54** |
| **C-10** | 57028202 | >50 | 210 ± 32 | <4 | >250 |
| **C-11** | 32422044 | >50 | >250 | >5 | >250 |
| **C-12** | 57993308 | >25 | >250 | >10 | >250 |
| **C-13** | 19272202 | >50 | 17.8 ± 3.1 | <1 | 173 ± 51 |
| **C-14** | 19370091 | >50 | >250 | >5 | 136 ± 70 |
| **C-15** | 19601035 | >50 | 520 ± 12 | >10 | 61.8 ± 27.0 |
| **C-16** | 25583081 | 20.3 ± 0.6 | 86.3 ± 8.5 | 4 | 169 ± 40 |
| **C-17** | 32376900 | >50 | >250 | >5 | >250 |
| **C-18** | 32377208 | >50 | >250 | >5 | 227 ± 5 |
| **C-19** | 32378407 | >50 | 220 ± 26 | <4 | 42.0 ± 9.5 |
| **C-20** | 20164181 | >50 | >250 | >5 | 77.7 ± 12.9 |
| **C-21** | 30858246 | >50 | >250 | >5 | 166 ± 22 |
| **C-22** | 56990017 | >50 | >250 | >5 | >250 |
| **C-23** | 20254711 | >50 | >250 | >5 | >250 |
| **C-24** | 56798609 | 10.9 ± 5.8 | 50.0 ± 14.1 | 5 | 81.7 ± 38.8 |
| **C-25** | 56797240 | >50 | >250 | >5 | >250 |
| **C-26** | 56864385 | >50 | >250 | >5 | >250 |
| **C-27** | 18700793 | >50 | >250 | >5 | >250 |
| **C-28** | 13784516 | 25.3 ± 1.5 | >250 | >10 | >250 |
| **C-29** | 56923915 | >50 | >500 | >10 | 56.3 ± 4.7 |
| **C-30** | **19598270** | **3.6 ± 2.1** | **>250** | **>69** | **183 ± 9** |

^a^ 50% Effective Concentration at half-maximal response, i.e., the compound concentration that inhibits 50% of plaque formation, as determined by PRAs against DENV-2 NGC strain in Vero cells. Reported values represent the means ± SD of data derived from n ≥ 3 independent experiments in duplicate.

^b^ 50% Cytotoxic concentration that produces 50% of cytotoxicity, as determined by MTT assays in Vero cells. Reported values represent the means ± SD of data derived from n = 3 independent experiments in duplicate.

^c^ SI, Selectivity Index (determined as the ratio between CC_50_ and EC_50_).

^d^ 50% Inhibitory Concentration, the compound concentration that inhibits 50% of NS3-NS5 interaction in vitro, as determined by ELISA assay. Reported values represent the means ± SD of data derived from n ≥ 3 independent experiments in duplicate.

Data related to hit compounds **C-9** and **C-30** are in bold.
