# Supplementary material for: Small-Molecule Inhibitor of Flaviviral NS3-NS5 Interaction with Broad-Spectrum Activity and Efficacy In Vivo
Source: mBio. 2023 Jan 9;14(1):e03097-22. doi: 10.1128/mbio.03097-22 (PMC9973282; doi:10.1128/mbio.03097-22)
Supplement: FIG S4 [file mbio.03097-22-s0004.docx]

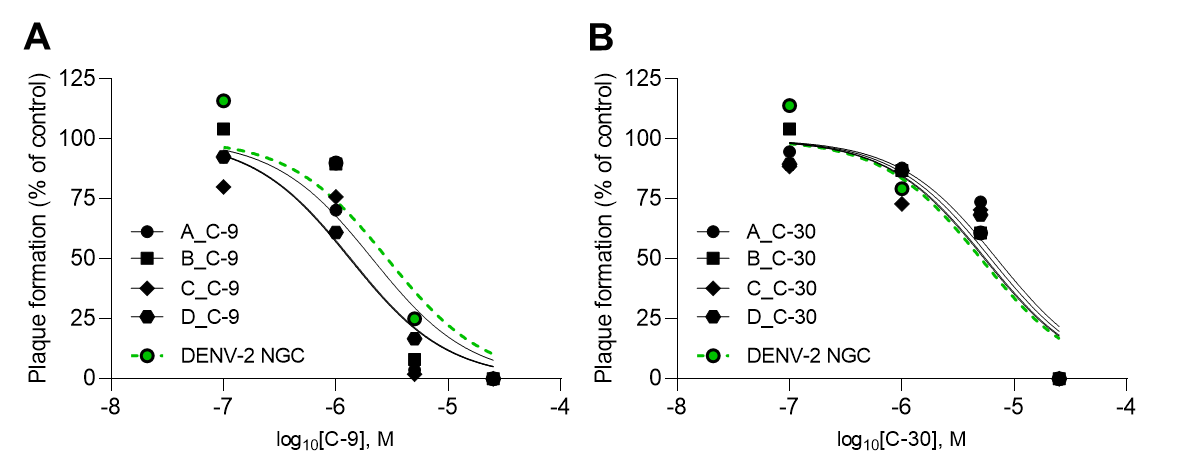


**Figure S4.** **Concentration-dependent inhibition of DENV-2 clones passaged in the presence of C-9 and C-30.** Plaque reduction assays were performed in Vero cells infected with either DENV-2 NGC P10 strain passaged in DMSO or different DENV-2 P10 viruses (clones A to D) extensively passaged in the presence of increasing concentrations of hit compounds (A) C-9 and (B) C-30 (please refer also to Table S2A). Vero cells were infected with the different viruses and then treated with increasing doses (from 0.1 to 25 µM) of the indicated test compounds. Graphs represent the mean of n = 2 independent experiments in duplicate.
