# Supplementary material for: Small-Molecule Inhibitor of Flaviviral NS3-NS5 Interaction with Broad-Spectrum Activity and Efficacy In Vivo
Source: mBio. 2023 Jan 9;14(1):e03097-22. doi: 10.1128/mbio.03097-22 (PMC9973282; doi:10.1128/mbio.03097-22)
Supplement: TABLE S2 [file mbio.03097-22-s0006.docx]

**Table S2 Additional supplementary information about the selection of resistant viruses, oligonucleotides, and antibodies.**

A. Scheme of the DENV-2 resistant strains isolation procedure

| **Passage** | **C-9 concentration (µM)** | **C-30 concentration (µM)** |
| --- | --- | --- |
| P1 | 5 | 7 |
| P2 | 10 | 15 |
| P3 | 20 | 25 |
| P4 | 25 | 25 |
| P5-P10 | 25 | 25 |

B. Oligonucleotides used in this study

| D2_NS3(177-618) | Primer FOR | TTAGAGGGATCCGAGATCGAAGATGAC |
| --- | --- | --- |
|  | Primer REV | TTAGAGAAGCTTTTACTTTCTTCCAGCTGC |
| D2_NS5(272-900) | Primer FOR | TTACACGGATCCACCCGCAACATCGGA |
|  | Primer REV | TTAGAGGTCGACCTACCACAGGACTCCTGC |

C. Antibodies used in this study

| **Antibody** | **Supplier** | **ID** | **Usage (Dilution)** |
| --- | --- | --- | --- |
| anti-DENV-2 NS5 | ThermoFisher | GT361 | E (1:3,000) |
| anti-Mouse IgG | Millipore | - | E (1:3,000) |
| anti-β-Actin | Sigma | AC-74 | WB (1:8,000) |
| anti-DENV-2 NS3 | ThermoFisher | GT2811 | WB (1:2,000) |
| anti-DENV-2 NS5 | Genetex | GTX124253 | WB (1:2,000) |
| anti-Mouse IgG (H+L) | Santa Cruz | - | WB (1:2,000) |
| anti-DENV-2 ENV | ThermoFisher | 5184 | IF (1:100) |
| anti-Mouse IgG (H+L) | Invitrogen | - | IF (1:2,000) |

Applications: E, ELISA; WB, Western Blot; IF, immunofluorescence.
