# Supplementary material for: Small-Molecule Inhibitor of Flaviviral NS3-NS5 Interaction with Broad-Spectrum Activity and Efficacy In Vivo
Source: mBio. 2023 Jan 9;14(1):e03097-22. doi: 10.1128/mbio.03097-22 (PMC9973282; doi:10.1128/mbio.03097-22)
Supplement: TABLE S3 [file mbio.03097-22-s0007.docx]

Table S3 Antiviral activity against different single-stranded positive RNA viruses

A. Antiviral Activity of selected hit compounds against ZIKV and WNV

| **Hit #** | **ZIKV**  **EC_50_^a^ (µM) - CI^b^** | **SI^c^** | **WNV**  **EC_50_^d^ (µM) - CI** | **SI** |
| --- | --- | --- | --- | --- |
| **C-1** | >50 | >5 | ND | - |
| **C-3** | 0.59 (0.26 – 1.29) | 71 | >25 | >1 |
| **C-4** | 14.0 (8.1 – 23.6) | 8 | >25 | >4 |
| **C-5** | >10 | <1 | ND | - |
| **C-6** | 12.3 (6.6 – 21.9) | 6 | ND | - |
| **C-8** | 19.3 (12.8 – 31.0) | 2 | ND | - |
| **C-9** | **2.1 (1.3 – 3.5)** | **119** | **1.7 (0.9 – 2.9)** | **>147** |
| **C-15** | >50 | 10 | ND | - |
| **C-24** | 0.6 (0.4 – 1.1) | 23 | 4.2 (2.2 – 7.5) | 7 |
| **C-29** | >50 | >10 | >25 | >20 |
| **C-30** | **1.6 (1.1– 2.4)** | **>156** | **1.97 (1.3 – 3.0)** | **>126** |

^a^ 50% Effective Concentration al half-maximal response, the compound concentration that inhibits 50% of plaque formation, as determined by PRAs against ZIKV PRVABC strain in Vero cells. Reported values derived from n ≥ 3 independent experiments in duplicate.

^b^ CI, Confidence Interval (95% Profile likelihood, calculated with GraphPad Prism 8.0 software).

^c^ SI, Selectivity Index (determined as the ratio between CC_50_ and EC_50_).

^d^ 50% Effective Concentration at half-maximal response, the compound concentration that inhibits 50% of plaque formation, as determined by PRAs against WNV NY99 strain in Vero cells. Reported values represent derived from n ≥ 3 independent experiments in duplicate.

ND: Not Determined. Data related to hit compounds **C-9** and **C-30** are in bold.

B. Antiviral activity of **C-9**, **C-30**, and reference compound **NITD-008** against different single-stranded positive RNA viruses

| **Virus** | **C-9** | | **C-30** | | **NITD-008** | |
| --- | --- | --- | --- | --- | --- | --- |
|  | **EC_50_^a^ (µM)** | **EC_90_^b^ (µM)** | **EC_50_ (µM)** | **EC_90_ (µM)** | **EC_50_ (µM)** | **EC_90_ (µM)** |
| **DENV-2** | 2.4 ± 1.8 | 23.4 ± 3.6 | 3.7 ± 2.0 | 39.8 ± 6.5 | 2.0 ± 0.5 | 17.4 ± 1.9 |
| **ZIKV** | 2.1 ± 0.8 | 17.8 ± 2.2 | 1.6 ± 0.7 | 12.0 ± 2.6 | 0.3 ± 0.1 | 3.4 ± 0.9 |
| **WNV** | 1.7 ± 0.9 | 14.1 ± 1.9 | 2.0 ± 0.7 | 41.7 ± 4.8 | 1.1 ± 0.6 | 8.9 ± 1.1 |
| **CHIKV** | >20 | >20 | >20 | >20 | ND | ND |
| **COXVB1** | >20 | >20 | >20 | >20 | ND | ND |

^a^ 50% Effective Concentration al half-maximal response, the compound concentration that inhibits 50% of plaque formation, as determined by PRAs against different viruses. Reported values derived from n ≥ 3 independent experiments in duplicate.

^b^ The compound concentration that inhibits 90% of plaque formation, as determined by PRAs against different viruses. Reported values derived from n ≥ 3 independent experiments in duplicate.

ND: Not Determined.
