# Supplementary material for: Small-Molecule Inhibitor of Flaviviral NS3-NS5 Interaction with Broad-Spectrum Activity and Efficacy In Vivo
Source: mBio. 2023 Jan 9;14(1):e03097-22. doi: 10.1128/mbio.03097-22 (PMC9973282; doi:10.1128/mbio.03097-22)
Supplement: TABLE S4 [file mbio.03097-22-s0008.docx]

**Table S4 Biological evaluation of commercially available analogs of hit compound C-9**

| **Hit #** | **ZINC CODE** | **Cytotoxicity**  **CC_50_^a^ (µM)** | **NS3-NS5**  **ELISA,**  **IC_50_^b^ (µM)** | **Antiviral Activity**  **EC_50_^c^ (µM)** | **Antiviral Activity**  **EC_50_^d^ (µM)** | **Antiviral**  **Activity**  **EC_50_^e^ (µM)** |
| --- | --- | --- | --- | --- | --- | --- |
| **C-31** | 01333417 | 45.2 ± 21.1 | >250 | >50 | ND | ND |
| **C-32** | 01333418 | 108 ± 36 | >250 | >50 | ND | ND |
| **C-33** | 01333425 | >500 | >250 | 3.8 ± 1.1 | 2.8 ± 1.5 | 2.5 ± 1.3 |
| **C-34** | 01333352 | 19.8 ± 5.5 | >250 | >50 | ND | ND |
| **C-35** | 01333376 | >500 | >250 | >50 | ND | ND |
| **C-36** | 01333365 | 58.8 ± 12.9 | 65.3 ± 18.7 | >50 | ND | ND |
| **C-37** | 01333354 | >500 | >250 | 44.1 ± 8.5 | ND | ND |
| **C-38** | 00922980 | 44.0 ± 13.0 | 56.2 ± 7.1 | >50 | ND | ND |
| **C-39** | 01333342 | >500 | 156 ± 35 | >50 | ND | ND |
| **C-40** | 01333401 | 30.3 ± 8.1 | 182 ± 37 | >50 | ND | ND |
| **C-41** | 01333349 | 28.8 ± 0.8 | >250 | 33.2 ± 7.8 | ND | ND |
| **C-42** | 00922979 | 105 ± 14 | 41.3 ± 2.5 | 7.6 ± 3.5 | 6.8 ± 1.4 | 9.8 ± 2.8 |
| **C-43** | 01379321 | 34.5 ± 16.2 | 148 ± 75 | >50 | ND | ND |
| **C-44** | 01333422 | >500 | >250 | >50 | ND | ND |

^a^ Compound concentration that produces 50% of cytotoxicity, as determined by MTT assays in Vero cells. Reported values represent the means ± SD of data derived from n = 3 independent experiments in duplicate.

^b^ 50% Inhibitory Concentration, the compound concentration that inhibits 50% of NS3-NS5 interaction *in vitro*, as determined by ELISA assay.

^c^ Effective Concentration at half-maximal response, i.e., the compound concentration that inhibits 50% of plaque formation, as determined by PRAs against DENV-2 NGC strain in Vero cells.

^d^ 50% Effective Concentration al half-maximal response, the compound concentration that inhibits 50% of plaque formation, as determined by PRAs against ZIKV PRVABC strain in Vero cells.

^e^ 50% Effective Concentration at half-maximal response, the compound concentration that inhibits 50% of plaque formation, as determined by PRAs against WNV NY99 strain in Vero cells.

In all columns, reported values represent the means ± SD of data derived from n ≥ 3 independent experiments in duplicate. ND, Not Determined.
