# Supplementary material for: Small-Molecule Inhibitor of Flaviviral NS3-NS5 Interaction with Broad-Spectrum Activity and Efficacy In Vivo
Source: mBio. 2023 Jan 9;14(1):e03097-22. doi: 10.1128/mbio.03097-22 (PMC9973282; doi:10.1128/mbio.03097-22)
Supplement: TEXT S1 [file mbio.03097-22-s0010.pdf]

CERTIFICATE OF ANALYSIS

ENAMINE Ltd.
78 Chervonotkatska street, 02660 Kyiv, Ukraine
Telephone: +380 44 537 32 18
Fax: +380 44 537 32 53
www.enamine.net

Date of release: 2021.04.09

Recommended retest date: 2025.07.08

Sample lot number: 2021-0444659

Description

Name:	2-{[4-amino-5-(4-methylphenyl)-4H-1,2,4-triazol-3-yl]sulfanyl}-N-{4-[phenyl(propan-2-yl)amino]phenyl}acetamide
Code(s):	Z19598270
Formula:	C26H28N6OS
Formula weight:	472.6051
Storage temperature, °C:	RT
CAS number:	727718-65-2

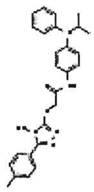

Analysis

Test	Specification	Result
State:	powder	powder
Colour:	lightbrown	lightbrown
Melting point, °C:	N/A	177
Purity (1H NMR)	95%	95%, conforms with structure, R2561608.WMF
LCMS	95%	100%, conforms with structure, R2561608.PDF

This product is intended for investigational laboratory use only. It is pharmaceutically unrefined, may contain traces of uncharacterized toxic impurities, and is not intended for use in humans. Responsibility for its use and compliance with all applicable laws rests solely with the purchaser.

I hereby certify that data shown in this certificate is correct and accurate.

Dr. A. Konovets,
Head of Quality Control Department

MaxPeak: 100.00%
Ret_Time: 1.389 min

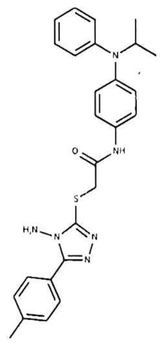

Mol Wt 472.6
Exact Mass 472.24

#	Time	Area%
1	1.389	100.00

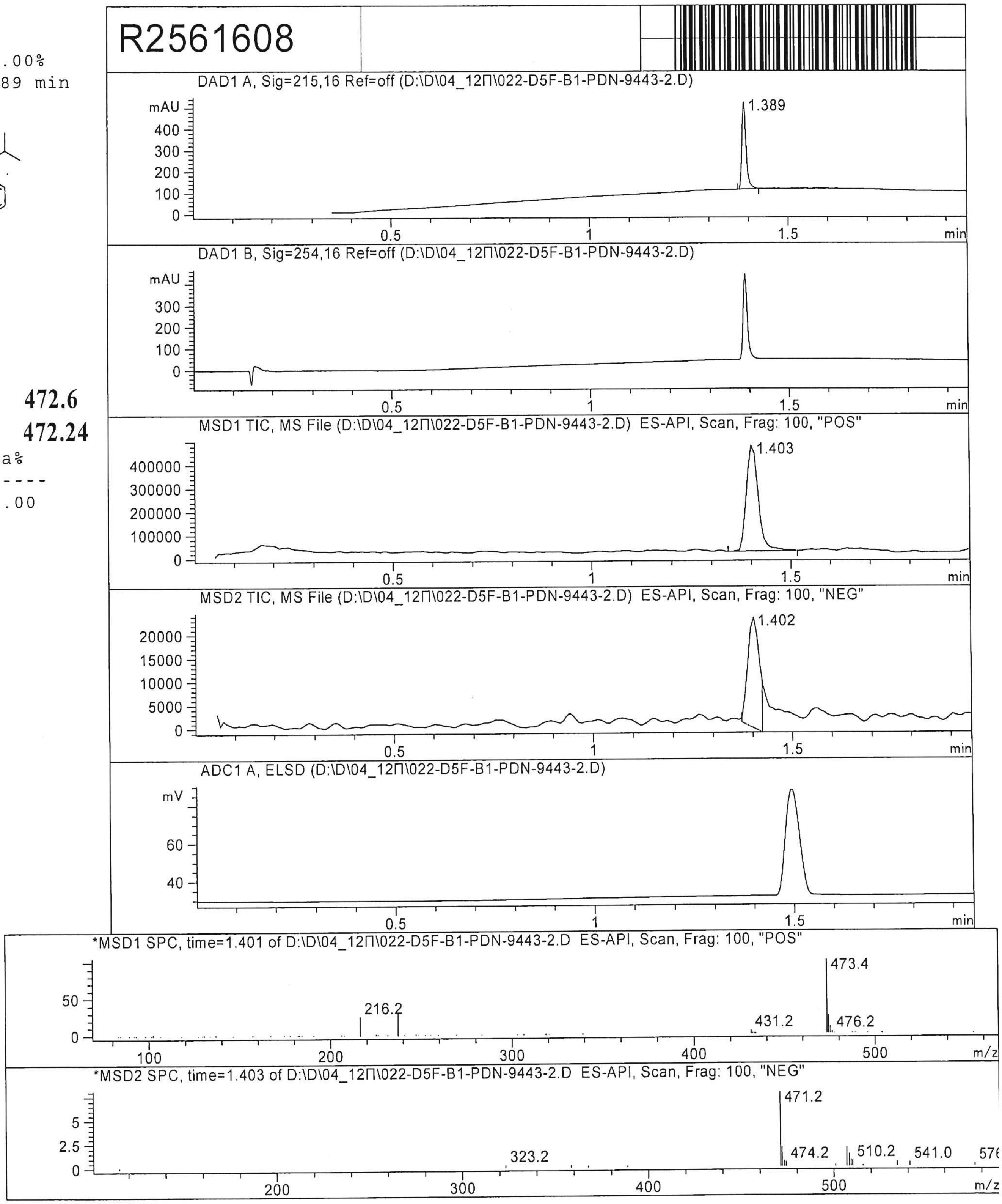

RT 1.403

RT 1.402

pdm-9443-2

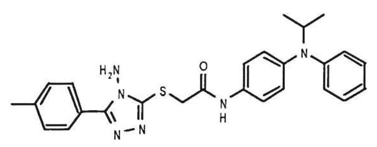

Z19598270

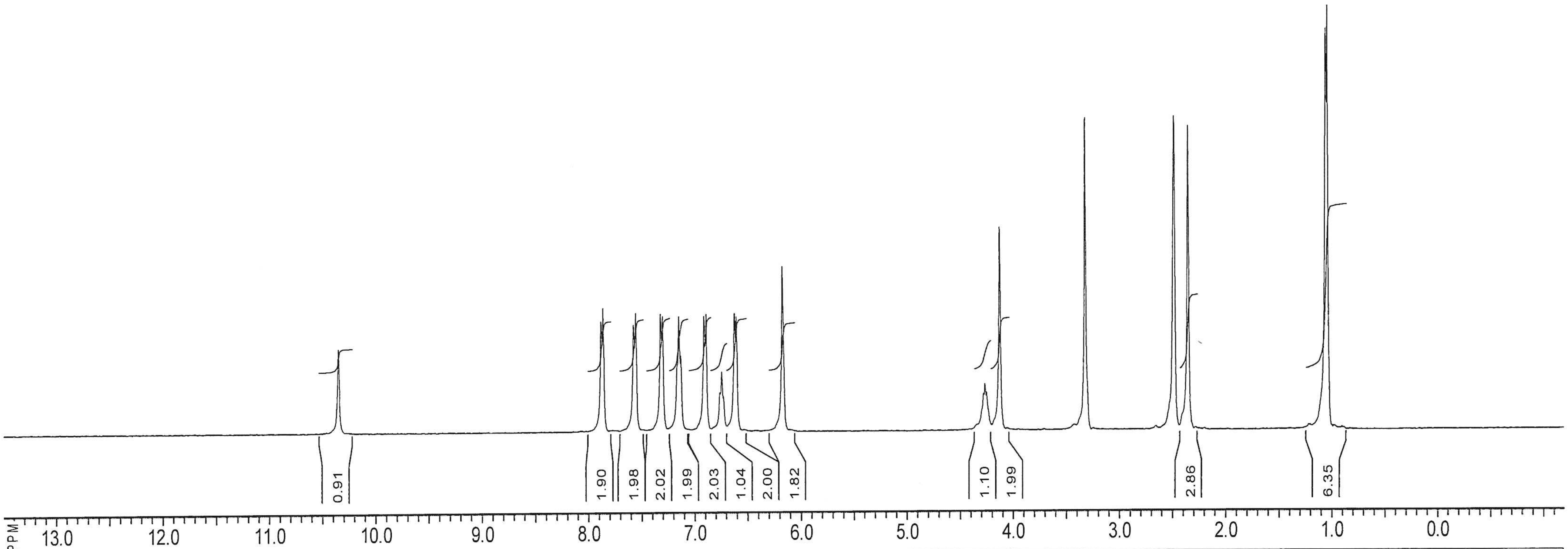

File name: pdm-9443-2	Operator: nmr	SF: 400.1300 MHz	NSC: 1	PW: 0.00 usec RG: 25	SI: 32768
